# AZD1222-induced nasal antibody responses are shaped by prior SARS-CoV-2 infection and correlate with virologic outcomes in breakthrough infection

**DOI:** 10.1016/j.xcrm.2022.100882

**Published:** 2022-12-15

**Authors:** Anastasia A. Aksyuk, Himanshu Bansal, Deidre Wilkins, Ann Marie Stanley, Stephanie Sproule, Jill Maaske, Satya Sanikommui, William R. Hartman, Magdalena E. Sobieszczyk, Ann R. Falsey, Elizabeth J. Kelly

**Affiliations:** 1 Translational Medicine, Vaccines & Immune Therapies, BioPharmaceuticals R&D, AstraZeneca, Gaithersburg, MD 20878, USA; 2 Biometrics, Vaccines & Immune Therapies, BioPharmaceuticals R&D, AstraZeneca, Gaithersburg, MD 20878, USA; 3 Clinical Development, Vaccines & Immune Therapies, BioPharmaceuticals R&D, AstraZeneca, Gaithersburg, MD 20878, USA; 4 Department of Anesthesiology, University of Wisconsin-Madison School of Medicine and Public Health, Madison, WI 53726, USA; 5 Division of Infectious Diseases, Department of Medicine, Vagelos College of Physicians and Surgeons, New York Presbyterian/Columbia University Irving Medical Center, New York, NY 10032, USA; 6 University of Rochester School of Medicine and Dentistry, Rochester, NY 14642, USA; 7 Rochester Regional Health, Rochester, NY 14621, USA

**Keywords:** AZD1222, ChAdOx1 nCoV-19, COVID-19 vaccine, SARS-CoV-2 spike antibodies, nasal antibody, nasal mucosal immunity, mucosal immune response, breakthrough infection, serology, immunoassay

## Abstract

The nasal mucosa is an important initial site of host defense against severe acute respiratory syndrome coronavirus 2 (SARS-CoV-2) infection. However, intramuscularly administered vaccines typically do not achieve high antibody titers in the nasal mucosa. We measure anti-SARS-CoV-2 spike immunoglobulin G (IgG) and IgA in nasal epithelial lining fluid (NELF) following intramuscular vaccination of 3,058 participants from the immunogenicity substudy of a phase 3, double-blind, placebo-controlled study of AZD1222 vaccination (ClinicalTrials.gov: NCT04516746). IgG is detected in NELF collected 14 days following the first AZD1222 vaccination. IgG levels increase with a second vaccination and exceed pre-existing levels in baseline-SARS-CoV-2-seropositive participants. Nasal IgG responses are durable and display strong correlations with serum IgG, suggesting serum-to-NELF transudation. AZD1222 induces short-lived increases to pre-existing nasal IgA levels in baseline-seropositive vaccinees. Vaccinees display a robust recall IgG response upon breakthrough infection, with overall magnitudes unaffected by time between vaccination and illness. Mucosal responses correlate with reduced viral loads and shorter durations of viral shedding in saliva.

## Highlights

• Anti-spike IgG is detected in nasal mucosa after intramuscular AZD1222 vaccination
• Nasal IgG correlated with serum IgG levels and showed durability through 1 year
• AZD1222 boosted existing anti-spike nasal IgA levels in seropositive individuals
• Nasal IgG levels correlated with reduced viral load and shedding duration in saliva

## Graphical abstract

**Figure:**
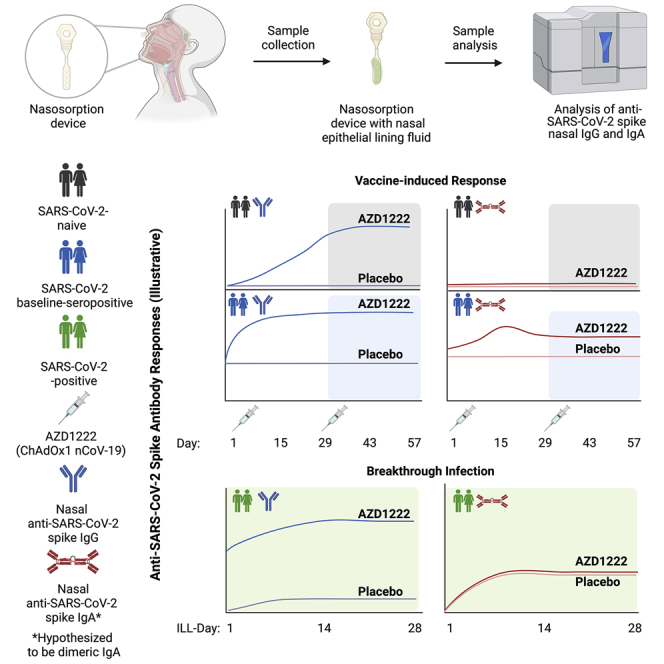

## Teaser

The nasal mucosa represents an important initial line of host defense against SARS-CoV-2. Aksyuk et al. describe anti-SARS-CoV-2 spike IgG and IgA responses in the nasal mucosa following intramuscular vaccination and upon breakthrough SARS-CoV-2 infection in participants from a phase 3, double-blind, placebo-controlled study of AZD1222 (ChAdOx1 nCoV-19) vaccination (ClinicalTrials.gov: NCT04516746).

## Introduction

The respiratory system is continuously exposed to microbes and thus relies on a robust mucosal immune response to maintain homeostasis against airborne respiratory pathogens.^1^ Severe acute respiratory syndrome coronavirus 2 (SARS-CoV-2) is a *Betacoronoavirus* that can produce severe and life-threatening respiratory pathologies in the absence of protection conferred by vaccination or immunoprophylaxis.^2^^,^^3^^,^^4^^,^^5^^,^^6^^,^^7^^,^^8^ The nasal epithelium is recognized as a portal for initial entry, infection, and transmission of SARS-CoV-2 because of its high levels of angiotensin-converting enzyme 2 receptor expression.^9^^,^^10^ Thus, the mucosa of the nasal cavity and resident innate and adaptive immune cells of the nasopharynx-associated lymphoid tissue (NALT) (also known as the Waldeyer’s ring in humans) comprise the first line of host defense against SARS-CoV-2 infection.^11^^,^^12^ Studies of individuals who have recovered from SARS-CoV-2 have suggested that impaired nasal epithelial anti-viral immunity may underlie and precede severe coronavirus disease 2019 (COVID-19).^13^ Furthermore, an effective humoral immune response in the nasal mucosa during the initial stages of SARS-CoV-2 infection has been associated with lower viral loads, reduced disease severity, and faster clinical symptom resolution.^14^

The ability to neutralize SARS-CoV-2 within the nasal mucosa prior to entry into the lower airways and lung is a highly desirable property for prophylactic therapy and may be particularly important for preventing transmission.^15^ Vaccines administered by intramuscular injection elicit antigen-specific systemic humoral and cell-mediated immune responses but are generally perceived as being incapable of generating protective mucosal immunity.^16^^,^^17^ AZD1222 (ChAdOx1 nCoV-19) is a simian, replication-deficient, adenovirus-vectored vaccine that is being used globally to mitigate the COVID-19 pandemic,^3^^,^^5^ with ∼2.5 billion doses administered in more than 170 countries within the first year of deployment.^18^ Two-dose primary series vaccination with AZD1222 has been observed to induce a robust polyfunctional Th1-biased cellular immune response^19^ and to elicit systemic anti-SARS-CoV-2 spike glycoprotein (anti-spike) and anti-receptor binding domain (anti-RBD) antibody responses.^3^^,^^20^^,^^21^^,^^22^ Serologic analyses have described additional anti-viral antibody functions following AZD1222 vaccination, including antibody-dependent neutrophil/monocyte phagocytosis, natural killer cell activation, and complement activation.

ClinicalTrials.gov: NCT04516746 is an ongoing phase 3 study of two-dose primary series AZD1222 vaccination administered with a 4-week interval.^23^ The first 3,058 participants who underwent randomization stratified by age group in the United States were recruited to a substudy to further assess the reactogenicity and immunogenicity of AZD1222. We have previously described strong anti-spike and neutralizing immunoglobulin G (IgG) antibody responses in this cohort during the double-blind, placebo-controlled portion of the study.^3^ Participants from this cohort were asked to provide nasal epithelial lining fluid (NELF) samples during study visits and throughout the follow-up period to assess immune responses in the nasal mucosa following vaccination. Study participants who developed protocol-defined COVID-19 symptoms were requested to contact their study site to initiate illness visits with additional NELF sample collection to assess the role of nasal immunity upon breakthrough SARS-CoV-2 infection.

Here we describe the presence of anti-spike IgG and IgA in NELF following intramuscular vaccination with AZD1222. We observed anti-spike IgG in NELF samples collected 14 days after the first dose of AZD1222 in SARS-CoV-2 infection-naive (baseline-seronegative) participants (defined by anti-SARS-CoV-2 nucleocapsid serology testing). IgG levels in NELF increased following a second dose of AZD1222, were durable, and persisted through at least 1 year after vaccination. AZD1222 vaccination also increased pre-existing anti-spike IgG and IgA responses in baseline-SARS-CoV-2-seropositive participants. IgG and IgA levels in NELF markedly increased upon breakthrough infection. Longer time intervals between vaccination and symptomatic illness did not affect the kinetics or magnitude of the NELF IgG response. Increased IgG levels in NELF correlated with reduced viral loads in saliva, with stronger correlations observed for vaccinees than placebo recipients. Increase in IgG and IgA levels in NELF correlated with shortened durations of viral shedding in saliva. Our findings are a detailed characterization of nasal IgG and IgA responses from a large-scale, placebo-controlled clinical study using an intramuscularly administered COVID-19 vaccine and are strengthened by the size and diversity of the immunogenicity substudy cohort. These data have implications for understanding how COVID-19 vaccines may mitigate SARS-CoV-2 transmission and will contribute to a broader understanding of the mechanisms by which intramuscularly administered vaccines confer protection from respiratory pathogens.

## Results

### Participants in the immunogenicity substudy

The first 1,527 participants aged 18–55 years, 769 participants aged 56–69 years, and 742 participants aged 70 years or older enrolled in the United States in ClinicalTrials.gov: NCT04516746 were recruited to the immunogenicity substudy (Table S1). Participants enrolled in the immunogenicity substudy provided NELF samples for the assessment of mucosal immune responses to vaccination during site visits on days 1, 15, 29, 43, 57, 180, and 360 (Figure S1). NELF samples were obtained prior to administration of AZD1222 or placebo on days 1 and 29. Immunogenicity data were windowed according to the timing of the first and second doses of AZD1222 or placebo to appropriately reflect the time point relative to the dosing days. Because of a clinical hold following an event of transverse myelitis in a different study of AZD1222,^5^ 775 substudy participants received their second dose of AZD1222 or placebo after a dosing interval longer than the planned 4 weeks (Table S2).

### Analysis of anti-spike IgG in immunogenicity substudy participant NELF following AZD1222 vaccination

**Figure 1:**
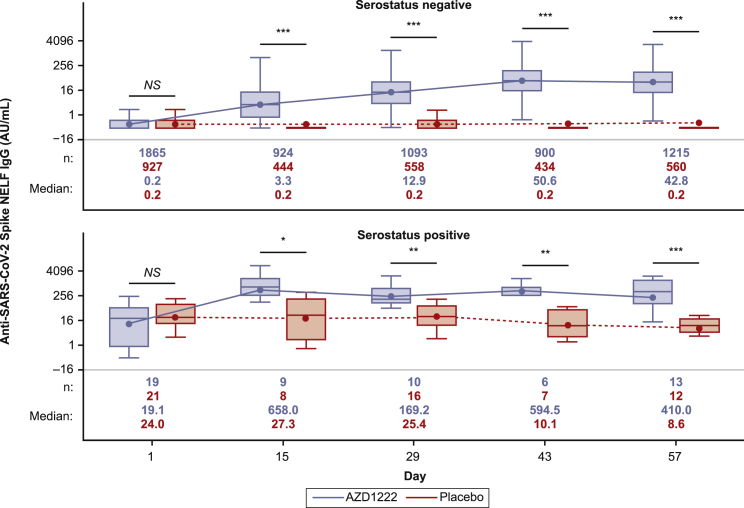
Quantification of anti-SARS-CoV-2 spike immunoglobulin G (IgG) in nasal epithelial lining fluid (NELF) from immunogenicity substudy participants following AZD1222 vaccination or placebo, by baseline serostatus Boxplots illustrate anti-SARS-CoV-2 spike IgG titers observed in NELF following AZD1222 vaccination or placebo according to participant baseline serostatus. Results are presented according to baseline SARS-CoV-2 serostatus as determined by the presence of SARS-CoV-2 nucleocapsid antibodies. The x axis denotes days since the first AZD1222 or placebo dose. Day 1 and day 29 samples were obtained prior to administration of AZD1222 or placebo. The box denotes interquartile range (IQR), the horizontal line in the box denotes median, and the marker in the box is the geometric mean titer (GMT). Any points more than 1.5 × IQR from the box were considered outliers and are not displayed. The whiskers that extend from the box indicate the minimum and maximum after removing the outliers. Boxplots were created using the log-normal distribution. To provide comprehensive information about the durability of immunogenicity after vaccination, data were censored in AZD1222 study participants at the time of non-study COVID-19 vaccination and for placebo participants at the earlier of the time of non-study COVID-19 vaccination or unblinding, whichever occurred first. Statistical evidence between groups was determined by post hoc two-tailed Mann-Whitney tests. Not significant (NS), p > 0.05; ∗p ≤ 0.05; ∗∗p ≤ 0.01; ∗∗∗p ≤ 0.001. AU/mL, arbitrary units per milliliter.

We assessed participant NELF for the presence of anti-spike antibodies using a qualified electrochemiluminescence-based serology assay.^24^ Levels of anti-spike IgG antibodies were low in NELF collected from baseline-seronegative participants prior to dosing and similar between the AZD1222 and placebo groups (Figure 1; Table S3). Baseline-seronegative participants had a marked increase in anti-spike IgG in NELF samples collected 14 days after the first dose of AZD1222. Levels of anti-spike IgG in NELF rose further after a second dose of AZD1222 and peaked on day 43, which is consistent with the kinetics of the anti-spike IgG response observed in serum (Table S3). IgG responses in NELF (defined as a ≥4-fold rise in geometric mean titers [GMTs] from baseline) were seen in 86% of baseline-seronegative participants in the AZD1222 group by day 29. Following the second dose of AZD1222, this figure rose to more than 96% on day 43 and day 57 (Table S3). Anti-spike IgG geometric mean fold rises (GMFRs) were lower in NELF compared with serum in samples from baseline-seronegative participants (GMFR of 104.30 and 380.46 on day 57 in NELF and serum, respectively; Table S3). Preliminary analyses indicate that IgG levels in NELF were maintained through day 180 and day 360 with some degree of expected waning (Figure S2).

In contrast to baseline-seronegative participants, samples from baseline-seropositive participants had approximately 95-fold higher levels of spike-specific IgG in NELF prior to AZD1222 vaccination. Although samples from baseline-seropositive participants had higher initial baseline titers, anti-spike IgG levels in NELF were nevertheless markedly increased on day 15. IgG levels were maintained through day 43 before starting to decline on day 57 (Figure 1). Long-term IgG responses in NELF from baseline-seropositive participants also displayed an expected degree of waning; however, the small number of these participants beyond day 57 limits interpretation (Figure S2).

Anti-spike IgG GMTs in NELF from vaccinated baseline-seronegative participants were comparable with baseline GMTs from baseline-seropositive placebo recipients from day 29 (11.99 and 11.49, respectively; Table S3) and were further increased after a second AZD1222 dose. Median IgG titers from vaccinated baseline-seronegative participants on day 360 were comparable with baseline titers in baseline-seropositive placebo recipients (18.3 versus 19.1; Figure S2).

### Evaluation of anti-spike IgA responses in immunogenicity substudy participant NELF following vaccination

**Figure 2:**
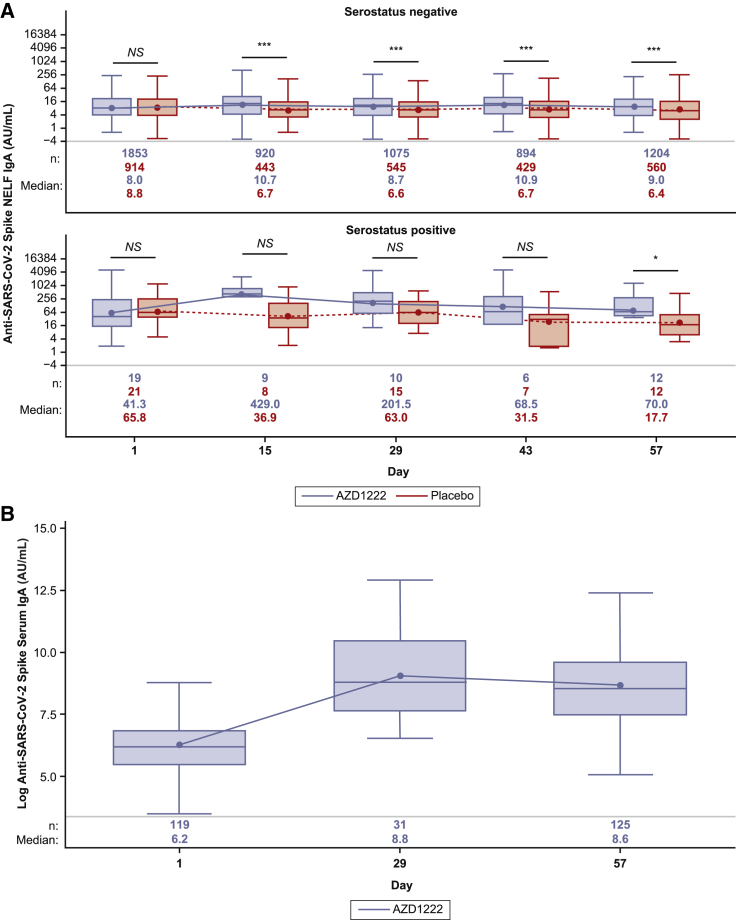
Quantification of anti-SARS-CoV-2 spike IgA in NELF and serum from immuno-genicity substudy participants following AZD1222 vaccination or placebo (A) Boxplots illustrating anti-SARS-CoV-2 spike IgA titers observed in NELF following AZD1222 vaccination or placebo according to participant baseline SARS-CoV-2 serostatus, as determined by the presence of SARS-CoV-2 nucleocapsid antibodies. To provide comprehensive information about the durability of immunogenicity after vaccination, data were censored in AZD1222 study participants at the time of non-study COVID-19 vaccination and for placebo participants at the earlier of the time of non-study COVID-19 vaccination or unblinding, whichever occurred first. (B) Post hoc analysis of IgA titers observed in serum of baseline-seronegative participants following AZD1222 vaccination. The x axis denotes days since the first AZD1222 or placebo dose. Day 1 and day 29 samples were obtained prior to administration of AZD1222 or placebo. The box denotes IQR, the horizontal line in the box denotes median, and the marker in the box is the GMT. Any points more than 1.5 × IQR from the box were considered outliers and are not displayed. The whiskers that extend from the box indicate the minimum and maximum after removing the outliers. Boxplots were created using the log-normal distribution. Data were censored in participants at the time of non-study COVID-19 vaccination during this post hoc analysis of baseline-seronegative AZD1222 vaccinees. Participants who tested positive for the presence of SARS-CoV-2 nucleocapsid antibodies at any time after day 1 were excluded from this analysis. Statistical evidence between groups was determined by post hoc two-tailed Mann-Whitney tests. NS, p > 0.05; ∗p ≤ 0.05; ∗∗p ≤ 0.01; ∗∗∗p ≤ 0.001.

In NELF samples from baseline-seronegative participants, vaccination with AZD1222 did not substantially increase anti-spike IgA titers, which remained similar to those in the placebo group after the first and second doses (Figure 2A; Table S3). Serum IgA levels were assessed in a subset of baseline-seronegative participants as part of a post hoc exploratory analysis after no increase in anti-spike IgA was observed in NELF following AZD1222 vaccination. AZD1222 vaccination induced a serum anti-spike IgA response that was maintained following a second dose (Figure 2B). A slight increase in anti-spike IgA was observed in NELF samples collected 14 days after the first dose of AZD1222 from baseline-seropositive participants (Figure 2A). In this population, median anti-spike IgA titers did not increase following a second AZD1222 dose and showed signs of initial decline, corresponding to the expected short half-life of IgA.

### Correlations between anti-spike IgG and IgA levels in immunogenicity substudy participant NELF and serum following AZD1222 vaccination

**Figure 3:**
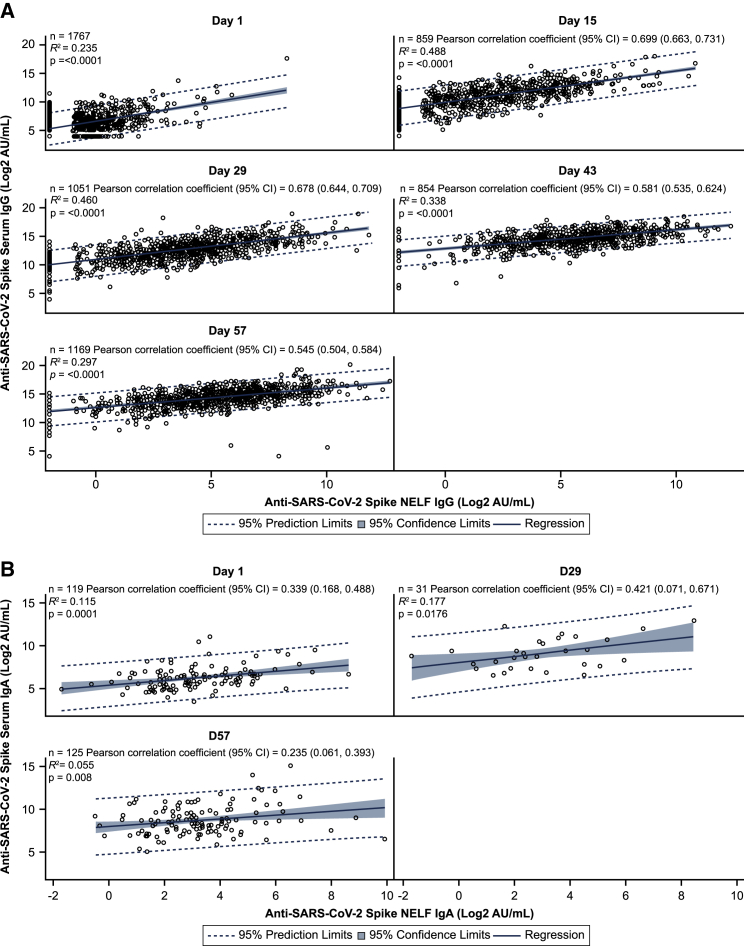
Analysis of anti-SARS-CoV-2 spike IgG and IgA levels in serum and NELF from immunogenicity substudy participants following AZD1222 vaccination (A) Post hoc correlation analysis depicting the relationship between serum anti-spike IgG levels (y axis) and nasal anti-spike IgG levels (x axis) in baseline-seronegative and baseline-seropositive immunogenicity substudy participants following AZD1222 vaccination. Blue shading denotes 95% confidence limits. A dotted line denotes 95% prediction limits. Clustering of participants along the y axis occurs because of levels of anti-SARS-CoV-2 spike IgG in NELF falling below the assay lower limit of quantification (LLOQ). Dilution-adjusted LLOQ SARS-CoV-2 spike IgG = 0.49 (AU/mL); upper limit of quantification (ULOQ) spike IgG = 7,000 AU/mL. (B) Post hoc correlation analysis depicting the relationship between serum (y axis) and nasal (x axis). IgA samples being compared for each participant are from samples obtained at the same visit. Dilution-adjusted LLOQ SARS-CoV-2 spike IgA = 0.62 (AU/mL); ULOQ spike IgA = 5,000 AU/mL. To provide comprehensive information about durability of immunogenicity after vaccination, data were censored in study participants at the time of receipt of the non-study COVID-19 vaccine, if applicable, but not at the time of unblinding. Participants who tested positive for the presence of SARS-CoV-2 nucleocapsid antibodies at any time after day 1 were excluded from this analysis. CI, confidence interval.

We evaluated correlations between the levels of anti-spike IgG and IgA in NELF and serum of baseline-seronegative and baseline-seropositive participants following AZD1222 vaccination. Anti-spike IgG levels in NELF and serum were well correlated at all post-baseline time points with Pearson correlation coefficients in the range of 0.545–0.699 (Figure 3A). Pre-existing anti-spike IgG immunity did not influence the strength of correlation because Pearson correlation coefficients were similar at all time points when baseline-seropositive participants were excluded from the analysis. Levels of anti-spike IgG displayed similar kinetics between NELF and serum samples (Table S3). Median serum-to-NELF IgG partition ratios (i.e., the proportion of serum IgG that transudates from serum to NELF) were estimated consistently at 1%–1.2% across days 15–57. The day 1 serum-to-NELF partition ratio was 4.5%, likely reflecting non-specific IgG transudation because of the very low levels of antigen-specific antibodies at baseline and lower correlations between serum and NELF measurements (Table 1). Serum spike-binding IgG antibodies (measured using a serology binding assay) displayed a high correlation with serum anti-SARS-CoV-2 neutralizing titers (Pearson correlation coefficients: 0.585–0.825) (Figure S3).

**Table 1:** Summary of serum: NELF IgG partition ratio (percent) using the median dilution (5.01) factor in baseline-seronegative participants following AZD1222 vaccination

Summary Statistics	Day 1	Day 15	Day 29	Day 43	Day 57
N	1,875	903	1,101	899	1,212
Partition ratio geometric mean	4.53	1.06	1.24	1.10	1.20
95% CI for geometric mean	(4.33, 4.75)	(0.97, 1.16)	(1.14, 1.35)	(1.00, 1.21)	(1.11, 1.30)
Geometric %CV	1.38	2.29	2.35	2.48	2.62
Min	0.05	0.01	0.01	0.00	0.00
Max	262.31	49.67	265.36	51.80	14,138.06

CI, confidence interval; CV, coefficient of variation; IgG, immunoglobulin G; Max, maximum; Min, minimum; NELF, nasal epithelial lining fluid.

In order to provide comprehensive information on durability of immunogenicity post vaccination, data was censored in study participants at time of receipt of non-study COVID-19 vaccine, if applicable, but not at time of unblinding.

Conversely, although monomeric serum IgA is also capable of transudation to NELF,^25^^,^^26^ the weak to moderate correlations observed at all post-baseline time points (Pearson correlation coefficients: 0.235–0.421) (Figure 3B) suggest that AZD1222 vaccination does not induce a sufficient serum IgA response to transudate to NELF. Anti-spike IgG and IgA levels in NELF displayed moderate correlation at all post-baseline time points in an analysis of all baseline-seronegative vaccinated participants (Pearson correlation coefficients: 0.455–0.485) (Figure S4).

### Analysis of anti-spike IgG and IgA levels in study participant NELF upon breakthrough SARS-CoV-2 infection

**Figure 4:**
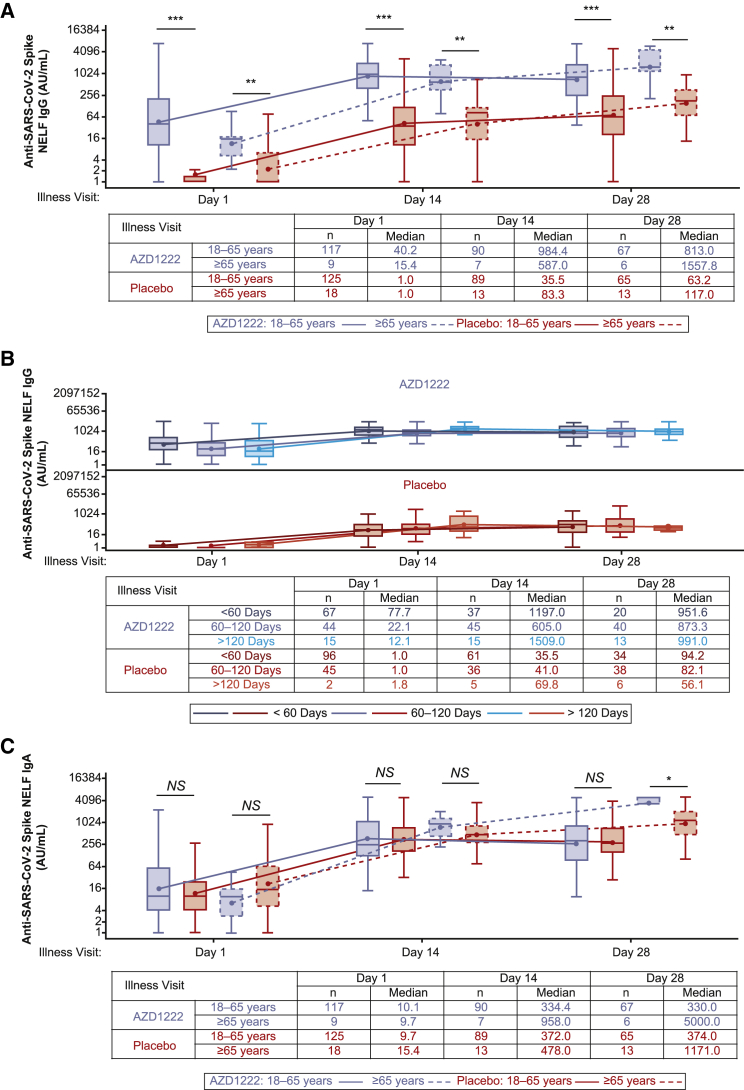
Quantification of anti-SARS-CoV-2 spike IgG and IgA levels in NELF from study participants with symptomatic breakthrough SARS-CoV-2 infection 15 or more days after the second AZD1222 vaccination or placebo (A–C) Boxplots illustrating anti-SARS-CoV-2 spike IgG (A and B) and IgA (C) titers observed in NELF obtained from baseline-seronegative study participants following reverse transcription polymerase chain reaction (RT-PCR)-positive symptomatic breakthrough SARS-CoV-2 infection 15 or more days after the second AZD1222 vaccination or placebo. Results are presented according to study age stratification (i.e., aged 18–65 years and ≥65 years) (A and C) or by time since the second dose primary series AZD1222 or placebo (i.e., <60 days, 60–120 days, and >120 days) (B). The x axis denotes days since the first illness visit for a period of 28 days. The box denotes IQR, the horizontal line in the box denotes median, and the marker in the box is the GMT. Any points more than 1.5 × IQR from the box were considered outliers and are not displayed. The whiskers that extend from the box indicate the minimum and maximum after removing the outliers. Boxplots are created using the log-normal distribution. IgA/G values between 0 and 1 are imputed as 1 to avoid negative log values. Participants who were unblinded or received non-study COVID-19 vaccination or exclusionary medication were excluded from this analysis. NELF sample results received after the database lock are included for samples collected up to the July 30, 2021 data cutoff. Results received after the database lock were not reconciled with the clinical database, and therefore updates to these data may be applied. Statistical evidence between groups was determined by post hoc two-tailed Mann-Whitney tests. NS, p > 0.05; ∗p ≤ 0.05; ∗∗p ≤ 0.01; ∗∗∗p ≤ 0.001.

We evaluated anti-spike IgG and IgA levels in NELF obtained from participants who experienced breakthrough SARS-CoV-2 infection more than 15 days after the second dose of AZD1222 or placebo (Table S4). Vaccinees demonstrated a robust recall IgG NELF response to AZD1222 with median titers that exceeded the response observed in placebo recipients at all time points (Figure 4A). The kinetics and magnitude of the NELF IgG response to breakthrough infection varied by age. Median NELF IgG titers peaked at illness visit day 14 (ILL-day 14) in vaccinees aged 18–65 years and at ILL-day 28 in vaccinees aged 65 years or older (Figure 4A). Similar differences in the kinetics and magnitude of the NELF IgG response were also observed by age within the placebo arm.

The kinetics and magnitude of NELF IgG responses to breakthrough infection were assessed by time intervals since the second dose of AZD1222 or placebo (Figure 4B). Lower absolute concentrations of IgG in NELF were observed at ILL-day 1 in those with longer intervals since primary series vaccination, consistent with expected immunological waning. Peak NELF IgG responses during the illness period were similar irrespective of the time since vaccination. No differences in the magnitude or kinetics of the NELF IgG response were observed between time interval subgroups within the placebo arm, as would be expected for a SARS-CoV-2 spike antigen-naive population.

The ILL-day 1 NELF IgA response to breakthrough infection was similar between vaccinees and placebo recipients (Figure 4C). Differences were observed between participant age groups. In participants aged 18–65 years, median IgA titers increased at ILL-day 14 and ILL-day 28 but remained similar between AZD1222 and placebo arms, while in vaccinees aged 65 years or older, median IgA titers continued to increase and were higher than placebo from ILL-days 14–28. The NELF IgA response was highest in individuals with less than 60 days since primary series vaccination and similar between individuals with 60–120 days or more than 120 days since primary series vaccination throughout the illness period (Figure S5). There were no changes in the magnitude or kinetics of NELF IgA response observed between time intervals within the placebo arm.

### Correlations between the IgG and IgA response in NELF and viral load and the duration of viral shedding in saliva upon breakthrough infection

**Figure 5:**
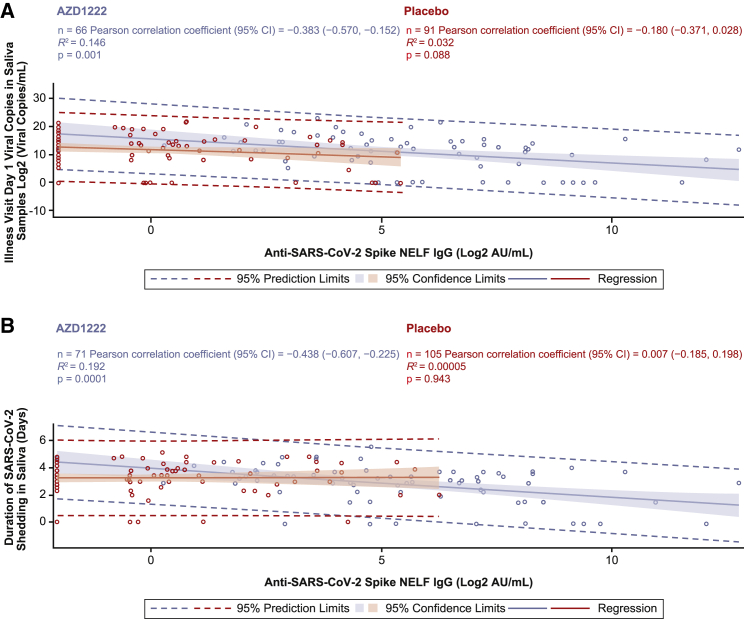
Analysis of ILL-day 1 anti-SARS-CoV-2 spike IgG levels in NELF versus ILL-day 1 viral load and duration of viral shedding in saliva samples from study participants with symptomatic breakthrough SARS-CoV-2 infection 15 or more days after AZD1222 primary series dose 2 or placebo (A and B) Post hoc correlation analyses depicting the relationship between ILL-day 1 anti-spike IgG levels in NELF (x axes) versus ILL-day 1 viral load in saliva samples (A) and duration of viral shedding in saliva samples (B) obtained from baseline-seronegative study participants with RT-PCR-positive symptomatic breakthrough SARS-CoV-2 infection 15 or more days after the second AZD1222 vaccination or placebo. Shading denotes 95% confidence limits. A dotted line denotes 95% prediction limits. Participants who were unblinded or received a non-study COVID-19 vaccination or exclusionary medication were excluded from this analysis. NELF sample results received after the database lock are included for samples collected up to the July 30, 2021 data cutoff. Results received after the database lock were not reconciled with the clinical database, and therefore updates to these data may be applied.

We assessed correlations between ILL-day 1 IgG in NELF and serum with SARS-CoV-2 viral load and the duration of viral shedding in saliva samples, as assessed by reverse-transcriptase polymerase chain reaction (RT-PCR) sample positivity. The ILL-day 1 IgG NELF response displayed a low to moderate negative correlation with viral load in saliva samples (Pearson correlation: AZD1222, −0.383; placebo, −0.180) and the duration of viral shedding (Pearson correlation: AZD1222, −0.438; placebo, 0.007), with stronger negative correlations observed in vaccinees than in placebo recipients (Figures 5A and 5B). ILL-day 1 IgG serum responses displayed similar low to moderate negative correlations with viral load in saliva samples (Pearson correlation: AZD1222, −0.436; placebo, −0.251) and the duration of viral shedding (Pearson correlation: AZD1222, −0.323; placebo, 0.067), with similar trends toward stronger correlations being observed in vaccinees compared with placebo recipients (Figure S6).

**Figure 6:**
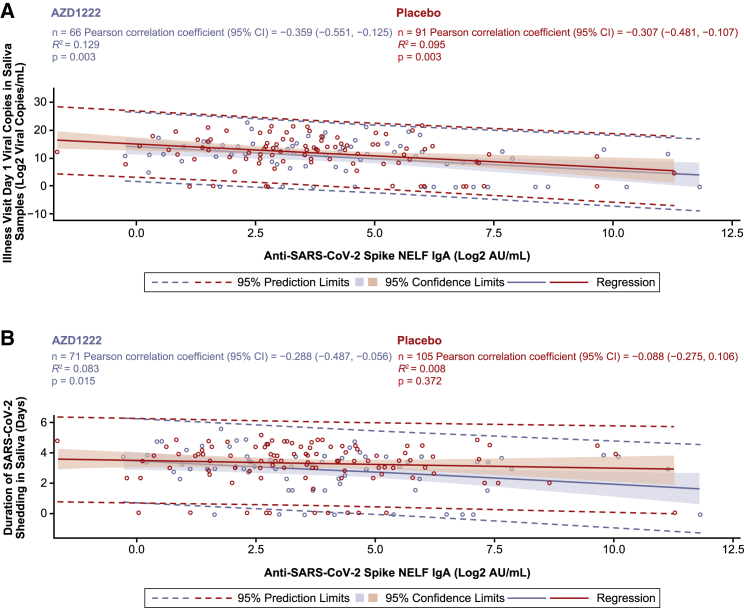
Analysis of ILL-day 1 anti-SARS-CoV-2 spike IgA levels in NELF versus ILL-day 1 viral load and duration of viral shedding in saliva samples from study participants with symptomatic breakthrough SARS-CoV-2 infection 15 or more days after AZD1222 primary series dose 2 or placebo (A–B) Post-hoc correlation analyses depicting the relationship between ILL-day 1 anti-spike IgA levels in NELF (x axes) versus ILL-day 1 viral load in saliva samples (A) and duration of viral shedding in saliva samples (B) obtained from baseline-seronegative study participants with RT-PCR-positive symptomatic breakthrough SARS-CoV-2 infection 15 or more days after the second AZD1222 vaccination or placebo. Shading denotes 95% confidence limits. A dotted line denotes 95% prediction limits. Participants who were unblinded or received a non-study COVID-19 vaccination or exclusionary medication were excluded from this analysis. NELF sample results received after the database lock are included for samples collected up to the July 30, 2021 data cutoff. Results received after the database lock were not reconciled with the clinical database, and therefore updates to these data may be applied.

ILL-day 1 IgA NELF responses also negatively correlated with viral loads in saliva samples (Pearson correlation: AZD1222, −0.359; placebo, −0.307) and the duration of viral shedding (Pearson correlation: AZD1222, −0.288; placebo, 0.088) (Figures 6A and 6B). Similar negative correlations were observed between vaccinee and placebo NELF IgA responses for viral load in saliva, while a moderate negative correlation was observed in vaccinees but not placebo recipients for duration of viral shedding.

## Discussion

Penetration into the upper airways of the respiratory tract is the first step of infection for many airborne diseases.^27^ Consequently, the resident antigen-presenting cells and T and B cells of the NALT are an important first line of host defense against many respiratory pathogens, including SARS-CoV-2.^11^^,^^12^^,^^28^ The NALT, like other mucosa-associated lymphoid tissues, functions as a two-tiered immunological barrier where intranasal or oral routes of vaccination elicit antigen-specific protective immunity in the mucosal and systemic immune compartments. This contrasts with intramuscular vaccination, which typically induces poor mucosal immune responses despite eliciting good systemic immunity because of anatomic compartmentalization.^29^

In this manuscript, we present evidence of anti-spike IgG and IgA antibody responses in NELF following intramuscular AZD1222 vaccination. Our findings are strengthened by the size and diversity of the immunogenicity substudy (N = 3,038), which included ∼25% adults aged 70 years or older. Our findings build on other studies that have reported an oronasal IgG and IgA response following intramuscular BNT162b2 vaccination with similar kinetics to AZD1222 vaccination; however, their interpretation has been limited by small sample sizes (e.g., N ≤ 100), age-restricted populations (e.g., health care workers aged 18–55 years), or limited follow-up (e.g., 2–4 weeks after the second dose).^30^^,^^31^^,^^32^^,^^33^^,^^34^ Additionally, the differences in the kinetics and magnitude of IgG and IgA responses between different participant populations (i.e., between baseline-SARS-CoV-2-seronegative and baseline-seropositive participants and between vaccinated and unvaccinated participants) in our dataset provide a unique perspective on the different types of immune response elicited by natural SARS-CoV-2 infection and/or adenovirus-based COVID-19 vaccination. These findings are important as we navigate the era of “hybrid immunity.”

In baseline-seronegative participants, AZD1222 vaccination led to a robust spike-specific IgG response in NELF from 14 days following initial vaccination that was further increased by a second dose and durable through 1 year after vaccination. This indicates that AZD1222 is responsible for inducing and maintaining a durable IgG-specific response to the SARS-CoV-2 spike protein antigen, as would be expected for an intramuscularly delivered vaccine. Anti-spike IgG responses in NELF displayed kinetics similar to serum anti-spike IgG levels, likely reflecting transudation from the serum to NELF rather than IgG production by local oronasal B cells, as seen with live attenuated influenza vaccines.^35^ The durability of SARS-CoV-2 immunity is a subject of intense interest because of the severity of public health measures necessitated by the early phases of the pandemic.^36^^,^^37^ Therefore, it is encouraging that median anti-spike IgG titers in baseline-seronegative participant NELF exceeded those observed at baseline in seropositive participants by day 29 and remained significantly above baseline through to 1 year after vaccination.

Initial AZD1222 vaccination induced an anti-spike IgA response in baseline-seronegative participant serum, which was maintained with a second AZD1222 dose but did not substantially increase anti-spike IgA levels in NELF. Anti-spike IgA has also been observed in serum following intramuscular vaccination with primary series BNT162b2, Gam-COVID-Vac, and mRNA-1273, with low levels detected at the oronasal mucosae.^30^^,^^38^^,^^39^ While monomeric serum IgA is capable of serum-to-NELF transudation,^25^^,^^26^ our data suggest that serum IgA is not induced in sufficient quantities following AZD1222 vaccination to transudate to NELF. Thus, we hypothesize that the increase observed in anti-spike IgA levels in baseline-seropositive individuals following AZD1222 vaccination is dimeric IgA secreted from existing NALT-resident B cells induced from prior natural infection, as seen previously with mRNA and other adenovirus-based vaccines.^34^^,^^38^

Breakthrough SARS-CoV-2 infections induced a robust IgG recall response in vaccinee NELF. ILL-day 1 median IgG titers in vaccinees aged 18–65 years were comparable with those observed on day 57 in baseline-seronegative vaccinees aged 18 years or older (ILL-day 1: 40.2 versus day 57: 42.8). Median NELF IgG titers further increased beyond peak levels observed in baseline-seropositive vaccinees aged 18 years or older by ILL-day 14 and ILL-day 28 (ILL-days 14–28: 984.4–813.0; days 15–57: 658.0–410.0). Despite a lower initial median titer at ILL-day 1, median IgG titers in vaccinees aged 65 years or older were comparable with levels seen in baseline-seropositive vaccinees by ILL-day 14–28 (587.0–1557.8). Importantly, additional analyses of NELF IgG responses by time since primary series vaccination demonstrated that immunological waning did not affect the overall magnitude or affect the kinetics of the NELF IgG recall response, with median titers being similar across all time interval subgroups assessed. This contrasts with the IgA response to breakthrough infection in NELF, where median titers remained similar between vaccinees and placebo recipients aged 18–65 at all time points. Median IgA titers in vaccinees were low on ILL-day 1 but increased substantially throughout the illness period.

Induction of oronasal anti-spike IgG, IgA, and IgM has been observed following natural SARS-CoV-2 infection, likely reflecting the barrier functions of the respiratory tract mucosae^14^^,^^33^^,^^34^^,^^40^^,^^41^^,^^42^ and activation of other NALT-resident lymphoid cells to facilitate Ig class switching via T cell-dependent and T cell-independent means.^43^ We and others have observed that vaccination can boost prior anti-spike IgG and IgA responses, possibly because of reactivation of NALT-resident memory B cells upon exposure to cross-reactive spike antigen.^34^^,^^38^ Because cleaved SARS-CoV-2 spike protein has been observed in vaccinee serum in preclinical and clinical settings, we speculate that some quantities of spike protein may reach the NALT following systemic injection, as previously hypothesized by Sano et al. (2022).^34^^,^^44^^,^^45^

These observations in individuals with prior spike-immunological memory contrast those in baseline-seronegative AZD1222 vaccinees, in whom IgA responses remained similar to those seen in placebo recipients at all time points. We hypothesize that the differences in antibody response between these populations arise because of the different routes of spike antigen exposure following vaccination versus natural infection. While natural infection has been observed to induce local respiratory tract-resident B cell and T cell responses,^46^^,^^47^ it appears that intramuscularly administered vaccines do not establish a similar NALT-resident immune memory in baseline-SARS-CoV-2-seronegative populations. These data are of interest in the context of a recent study comparing the immunogenicity of intramuscular and aerosolized forms of an adenovirus-vectored *Mycobacterium tuberculosis* (tuberculosis [TB]) vaccine, where the authors noted that, while the intramuscular and aerosolized forms induced robust systemic immune responses, only the aerosolized form induced lung-resident T cell responses.^48^ Increased levels of lung-resident T cell activation have similarly been observed with aerosolized versus intradermal forms of modified vaccinia Ankara-based TB vaccines.^49^^,^^50^ Collectively, these findings imply that antigen presentation within the respiratory tract may be required to instill sufficient NALT-resident immune memory. Taken together, these observations suggest that the IgA response we observed in the setting of breakthrough infection is a *de novo* local immune response rather than an anamnestic response and that different approaches (e.g., use of adjuvants, different routes of administration) will be required to improve mucosal immunogenicity for intramuscularly injected vaccines.^51^ We speculate, based on previous observations^34^^,^^38^^,^^52^ and our data in baseline-seropositive vaccinees, that subsequent AZD1222 vaccinations could boost any oronasal IgA immunity established by breakthrough infection.

Early studies of breakthrough infection have sought to characterize the roles of specific oronasal antibody responses in pursuit of a SARS-CoV-2 correlate of protection. Evidence suggests that early neutralizing responses are dominated by secretory IgA^25^ and that secretory IgA levels are inversely correlates with susceptibility to breakthrough infection following vaccination.^33^^,^^53^^,^^54^ There is comparatively little known about the specific protective role of transudated IgG in the upper respiratory tract during SARS-CoV-2 infection. Insights from preclinical and observational studies of respiratory syncytial virus and influenza virus suggest that transudated IgG neutralizes the host-derived virus in the respiratory tract after infection has been initiated, reducing viral loads and viral shedding and preventing severe disease by restricting the trajectory of infection.^35^^,^^55^^,^^56^^,^^57^ During an earlier clinical study, it was noted that AZD1222 plays a similar protective role at the nasal mucosa; decreased duration of SARS-CoV-2 PCR positivity from nasopharynx swabs and overall lower viral loads were observed in vaccinees with breakthrough infections.^58^ Similarly, others have observed an association between the respiratory tract IgG response and a reduction in levels of infectious virus in BNT162b2 vaccinees with breakthrough infections.^59^^,^^60^ Here we demonstrate that recall NELF IgG response following AZD1222 vaccination correlates with lower viral loads in vaccinee saliva. Although a recent preclinical study suggested that intramuscular AZD1222 vaccination elicits low levels of SARS-CoV-2 neutralizing IgG antibodies in the respiratory tract,^61^ our observations suggest that respiratory tract IgG could support SARS-CoV-2 clearance through other anti-viral functions, such as promoting activation of lung-resident phagocytes via opsonization, natural killer cell-mediated antibody-dependent cytotoxicity, and complement-dependent cytotoxicity, as observed in serum following primary series AZD1222 vaccination.^62^^,^^63^ NELF and serum IgG responses displayed similar degrees of negative correlations with viral outcomes, further supporting our hypothesis that serum IgG transudates to NELF. The NELF IgG and IgA response also correlated with a decreased duration of viral shedding in saliva at breakthrough infection. Collectively, these data provide important insight into the mechanisms by which intramuscularly administered COVID-19 vaccines influence transmission and confer protection against severe disease.

### Limitations of the study

ClinicalTrials.gov: NCT04516746 was designed as a double-blind, placebo-controlled study. In this substudy, participants could be unblinded and receive a non-study COVID-19 vaccination following US Food and Drug Administration (FDA) emergency use authorizations. Data were censored in AZD1222 study participants at the time of non-study COVID-19 vaccination and for placebo participants at the earlier of the time of non-study COVID-19 vaccination or unblinding, whichever occurred first. As described by Sobieszczyk et al.,^64^ despite our efforts to capture this information, there is evidence of an effect of under-reporting of non-study COVID-19 vaccination in the placebo arm on day 180 and day 360, which limits data interpretation beyond the day 57 time point, particularly for baseline-seropositive participants because of low participant numbers (Figure S2; Table S6). Participants aged 65 years or older were among the first eligible to receive non-study mRNA COVID-19 vaccinations, which may in part explain the increases we observed by participant age (Figures 4A and 4C). Another limitation of the study was associated with its initiation in August 2020; regrettably, the logistical challenges caused by the early phases of the pandemic impacted the availability of the synthetic absorption matrix devices used for NELF sample collection, thus reducing the number of samples available at the day 15, 29, 43, and 57 visits (Figure 1, Figure 2, Figure 3).

The long-term nasal IgG immunogenicity data (Figure S2) were generated from a preliminary analysis for an early assessment of the long-term immunogenicity up to day 360. These data were provided after the 6-month clinical database lock and therefore have not undergone full reconciliation against the clinical database. NELF sample results received after the database lock are included for samples collected up to the July 30, 2021, data cutoff used for the clinical database (Figure 4, Figure 5, Figure 6 and S5).

While one of the strengths of our immunogenicity substudy is its large sample size, data for our baseline-seropositive participants should not be overinterpreted because of the limited number of these participants enrolled in the substudy (n = 42). Further studies in baseline-seropositive individuals are needed to ascertain the wider effects of AZD1222 vaccination on existing responses to natural infection.

There remains an unmet need for a method of standardizing NELF collection. Therefore, there is inherent variation between studies of nasal mucosal immunity because of different sample collection methods (e.g., nasal wash, flocked swabs, and synthetic absorption matrix [SAM] strips) and normalization steps required for IgA.^65^ We were unable to determine IgG and IgA neutralizing antibody titers for this analysis because of the low volumes of NELF collected from participants and the subsequent dilution required during sample processing.

IgA is among the most heterogeneous of Igs and exists in secretory, polymeric, and monomeric forms. The assay to quantitatively assess mucosal IgA was developed and validated through assessments of precision, accuracy, and dilutional linearity; establishment of lower and upper limits of quantification [LLOQ and ULOQ, respectively]; and evaluation of stability for SARS-CoV-2 spike, RBD, and nucleocapsid antigens. Because this assay utilized an antibody that recognizes monomeric and dimeric IgA, we were unable to distinguish between different forms of IgA in this analysis.

To date, studies of the mucosal oronasal immune response following COVID-19 vaccination have been restricted to observational studies from small, single-center study populations. Our findings from a United States-wide, multicenter immunogenicity substudy conclusively demonstrate that two doses of intramuscularly administered AZD1222 induce a durable nasal anti-spike IgG response and can increase nasal IgA immunity from prior infection. Additionally, we demonstrate that AZD1222 vaccination produces a robust recall NELF IgG response upon breakthrough infection that correlates with reduced viral loads and, alongside NELF IgA, a reduced duration of viral shedding in vaccinees compared with placebo. Of note, breakthrough infections occurred at a time when the majority of cases were ancestral SARS-CoV-2.^64^

While vaccination has substantially reduced the global COVID-19 morbidity and mortality burden, current COVID-19 vaccines do not fully control transmission, and, consequently, the global prevalence of SARS-CoV-2 remains high. There has been intense interest in developing intranasal COVID-19 vaccines following observations of reduced viral shedding and complete upper respiratory tract protection in preclinical models of intranasal vaccination and subsequent SARS-CoV-2 challenge.^66^^,^^67^^,^^68^^,^^69^ Although a recent study using an intranasal formulation of AZD1222 failed to induce consistent antibody responses at the nasal mucosa, the authors acknowledged that the formulation/device combination used in the study was selected because it offered the prospect of rapid deployment during the peak period of the COVID-19 pandemic and that other formulations using, e.g., higher numbers of viral particles or including adjuvants could be assessed to improve the immunogenicity of intranasally administered adenovirus-vectored vaccines.^70^ These data, alongside further studies of the implications of effective immune responses in the nasal mucosa on breakthrough infection, will inform our understanding of immunity to SARS-CoV-2 and will have ramifications for the design of future vaccines against respiratory pathogens.

## Data sharing

Data underlying the findings described in this manuscript may be obtained in accordance with AstraZeneca’s data sharing policy described at https://astrazenecagrouptrials.pharmacm.com/ST/Submission/Disclosure.

## STAR★Methods

### Key resources table

**Table:** 

REAGENT or RESOURCE	SOURCE	IDENTIFIER
**Antibodies**		

SARS-CoV-2 IgG Panel Kit 2	Meso Scale Discovery, Rockville, MD, USA	K15383U
SULFO-TAG Anti-Human IgG Antibody	Meso Scale Discovery, Rockville, MD, USA	D21ADF
SARS-CoV-2 IgA Panel Kit 2	Meso Scale Discovery, Rockville, MD, USA	K15385U
SULFO-TAG Anti-Human IgA Antibody	Meso Scale Discovery, Rockville, MD, USA	D21ADE

**Critical commercial assays**		

Roche Elecsys Anti-SARS-CoV-2 serology immunoassay	F. Hoffmann-La Roche, Basel, Switzerland	09203095501

**Software and algorithms**		

Discovery Bench 4.0	Meso Scale Discovery, Rockville, MD, USA	Immunoassay Analysis Software | Meso Scale Discovery
SAS 9.4 Enterprise Guide 8.2.	SAS: Analytics, Artificial Intelligence and Data Management, Wittington House,Henley Road, Medmenham Marlow, Buckinghamshire, England, SL7 2EB	SAS/STAT 15.1, SAS/IML 15.1, SAS/GRAPH 9.4_M6

**Other**		

Nasosorption FX-I	Mucosal Diagnostics Ltd. Unit 17, Holmbush Industrial Estate, Midhurst, West Sussex, England, GU29 9HX	NSFL-FXI-IF10
SARS-CoV-2 Assay Plate 2	Meso Scale Discovery, Rockville, MD, USA	N05380A
Reference Standard 1	Meso Scale Discovery, Rockville, MD, USA	C00ADK
MSD Blocker A Kit	Meso Scale Discovery, Rockville, MD, USA	R93AA
Diluent 100	Meso Scale Discovery, Rockville, MD, USA	R50AA
MSD Wash Buffer (20X)	Meso Scale Discovery, Rockville, MD, USA	R61AA
MSD GOLD Read Buffer B	Meso Scale Discovery, Rockville, MD, USA	R60AM
Serology Control Pack 1	Meso Scale Discovery, Rockville, MD, USA	C4381

### Resource availability

#### Lead contact

Further information and requests for resources and reagents should be directed to and will be fulfilled by the lead contact, Elizabeth J. Kelly, PhD. (beth.kelly@astrazeneca.com)

#### Materials availability

This study did not generate any unique new reagents.

### Experimental model and subject details

#### Study participants

NCT04516746 is an ongoing phase 3 study assessing the safety, immunogenicity, and efficacy of AZD1222 for the prevention of symptomatic COVID-19 in participants ≥18 years of age whose conditions were medically stable and who were at increased risk for SARS-CoV-2 infection, including high risk for symptomatic and severe COVID-19.^3^^,^^23^^,^^64^ Participants were recruited from 88 sites in the United States, Chile, and Peru.

#### Study approval

The NCT04516746 study protocol and amendments were approved by the ethics committee or institutional review board at each participating center. The final version of the study protocol and statistical analysis plan have been published previously and can be accessed as part of (Sobieszczyk et al. 2022).^64^ The study was conducted in accordance with the principles of the Declaration of Helsinki and the International Council for Harmonization Good Clinical Practice guidelines. All participants provided written informed consent before enrollment.

#### Study design

NCT04516746 was designed as a double-blind, placebo-controlled study. Participants were randomly assigned to AZD1222 or placebo in a 2:1 ratio.^3^^,^^23^^,^^64^ Randomization was stratified according to age (≥18–65 years and ≥65 years), with a target of 25% or more of the participants being ≥65 years of age. Participants received two intramuscular injections of either AZD1222 (5×10^10^ viral particles), or saline placebo administered 4 weeks apart on days 1 and 29 (−3 to +7 days). Day 1 and day 29 NELF and blood samples were taken from all participants prior to the administration of AZD1222 or placebo. Immunogenicity substudy participants completed symptom diaries after vaccination and provided additional NELF and blood samples on days 15, 43, and 57. Immunogenicity data were windowed according to the timing of the first and second AZD1222 or placebo doses to appropriately reflect the timepoint relative to the dosing days.

All participants will remain in the study for 2 years (730 days) after receipt of the first dose of AZD1222 or placebo for safety follow-up and assessment of durability of immune response. For ethical reasons, participants in this substudy could be unblinded and receive non-study COVID-19 vaccination once available through US FDA emergency-use authorizations. The censoring implications of allowing non-study COVID-19 vaccinations are outlined in quantification and statistical analysis section.

### Method details

#### Baseline serostatus

Serostatus at baseline was defined by SARS-CoV-2 nucleocapsid antibody level as measured by Roche Elecsys Anti-SARS-CoV-2 serology test.

#### Illness visits

Study participants who experienced any duration of fever, shortness of breath or difficulty breathing, or chills, cough, fatigue, muscle aches, body aches, headache, new loss of taste, new loss of smell, sore throat, congestion, runny nose, nausea, vomiting, or diarrhea lasting ≥2 days were requested to contact their study site for confirmatory SARS-CoV-2 RT-PCR testing and to initiate illness visits with collection of nasopharyngeal swabs, saliva (US-sites only), and NELF samples for analysis.^3^

Only participants who had RT-PCR-confirmed SARS-CoV-2 infection were invited to complete the full 28-day illness visit course with additional site visits/sample collection on illness visit days 14, 21, and 28. If a participant had multiple sets of illness visits, the first set of illness visits with positive RT-PCR test result was used for the analysis. Breakthrough infection was defined as symptomatic RT-PCR-confirmed SARS-CoV-2 infection ≥15 days following the second dose primary series AZD1222 or placebo.^3^

#### Sample collection and processing

Participant serum was allowed to clot for 30 min at room temperature. Serum samples were centrifuged at 1,300xg for 15 min at room temperature within 1 h of collection. Cleared serum was stored at −70⁰C prior to analysis.

NELF was sampled during hospital visits using the Nasosorption FX-i (Mucosal diagnostics NSFL-FXI-IF10), a single-use device consisting of a synthetic absorption matrix (SAM) attached to an applicator handle designed to gently capture participant nasal mucosal lining fluid. The Nasosorption FX-i device was inserted into a participant’s nasal cavity by their healthcare practitioner. The participant was then asked to hold their nostril closed for 60 s before the device was returned to the collection tube. The SAM was stored at ≤–70⁰C prior to the elution of NELF. Once thawed, the SAM was removed from the Nasosorption FX-i collection tube using forceps. NELF was eluted by incubating the SAM with 330 μL of Diluent 100 (MSD R50AA) for 5–10 min at room temperature. Samples were centrifuged at 16,000xg for 15 min at room temperature and collected via Corning Spin-X centrifuge tube filters without membrane (Corning 9301) into Corning 2 mL polypropylene microcentrifuge tubes (Corning 3213). Samples were stored at ≤–70⁰C prior to analysis.

#### MSD® multiplex electrochemiluminescence serology assay

Participant NELF samples were evaluated for the presence of anti-SARS-CoV-2 spike antibodies using SARS-CoV-2 Plate 2 Kit (MSD N05380A) with IgG (MSD K15383U) and IgA (MSD K15385U) detection antibodies. The assay was qualified by MesoScale Discovery (MSD), which included determination of the ULOQ and LLOQ for the assay. Participant samples were pre-diluted 1:10- and 1:100-fold using MSD Diluent 100 solution.

Assay plates were blocked with 150 μL/well of blocking solution A (MSD R93AA) for 30 min at room temperature with shaking. Plates were washed three times with 1x wash buffer (MSD R61AA) prior to the addition of 50 μL/well of reference standard (MSD C00ADK), serology controls (MSD C4381), or diluted samples. Plates were sealed and incubated for 2 h at room temperature with shaking. Plates were washed three times with 1x wash buffer prior to the addition of 50 μL/well of 1x detection antibody solution (MSD D21ADF [IgG] D21ADE [IgA]) for 1 h at room temperature with shaking. Plates were washed with 1x wash buffer for a final three times and analyzed immediately following the addition of 150 μL/well of MSD GOLD Read Buffer B (MSD R60AM).

The sample concentration was determined by back-fitting the electrochemiluminescence signal to the MSD reference standard curve (MSD C00ADK) and reported in arbitrary units per milliliter (AU/mL). 1:100-dilution of the sample was reported, unless the value was below the LLOQ, in which case 1:10-dilution of the sample was reported.

Serum IgG samples were tested at PPD® in a validated assay using the MSD assay components as outlined in (Wilkins et al. 2022).^24^ Nasal IgG and serum IgG were measured in the same MSD electrochemiluminescence assay but using different reference standards. The conversion factor from MSD reference AU/mL (NELF IgG) and AZ AU/mL (Serum IgG) were determined previously.^24^

#### Determination of IgG serum-to-NELF partition ratio

The dilution of participant NELF during the sample elution step can be corrected by determining the dilution factor based on the differences in urea concentration between diluted NELF and undiluted serum samples. Unfortunately, urea concentration was not measured in NELF samples during this study. However, based on data supporting another investigation using the Nasosorption FX-i device, a median dilution factor of 5-fold,with respective first and third quartiles of 3- and 8-fold (Table S5) (n = 50) was used to estimate the serum-to-NELF partition ratio in this analysis.^71^

#### Pseudovirus neutralization assay

As outlined in (Falsey et al. 2021; Sobieszczyk et al. 2022),^3^^,^^64^ neutralizing antibodies in sera were assessed in a validated lentivirus-based SARS-CoV-2 pseudovirus assay (Monogram Biosciences, South San Francisco, CA). Pseudovirions containing luciferase and an ancestral SARS-CoV-2 virus spike protein were preincubated with serial dilutions of serum. Antibody titers are reported as the reciprocal of the serum dilution conferring 50% inhibition (ID_50_) of pseudovirus infection. A specificity control containing a non-specific pseudovirus (e.g., Avian Influenza envelope) was utilized to determine activity specific to SARS-CoV-2. Method validation included accuracy, repeatability, intermediate precision, and linearity.

#### Virologic assessments

As previously described (Falsey et al. 2021; Sobieszczyk et al. 2022),^3^^,^^64^ viral load (viral genome copies/mL) and viral shedding were assessed in saliva samples (collected at site illness visits or self-collected at home) using a validated RT-PCR assay for the quantitative measurement of SARS-CoV-2.

### Quantification and statistical analysis

The immunogenicity substudy population comprised the first 3,000 individuals enrolled at sites in the US. Participants were excluded from the immunogenicity population if they had important protocol deviations that were considered exclusionary for the population (detailed in full in^3^ supplemental material) judged to have the potential to interfere with the generation or interpretation of an immune response. Participants who received a prohibited medication were excluded from timepoints after the medication start date.

Due to the availability of non-study COVID-19 vaccines following emergency use authorization (EUA) during the study, participants were initially censored for all immunogenicity endpoints at the date of unblinding, or receipt of non-study COVID-19 vaccination, whichever occurred first, such that data from all subsequent visits was excluded from derivations and all by-visit summaries to provide comprehensive information on durability of immunogenicity post-vaccination. Censoring criteria were revised following the observation of increasing levels of anti-SARS-CoV-2 spike-binding and neutralizing antibodies on the placebo arm.^64^ Under the revised criteria, participants in both study arms were censored at the earliest date of non-study COVID-19 vaccination, excluding date of unblinding for the AZD1222 arm, and including the date of unblinding for placebo, whichever occurred first, with the aim of excluding effects of unreported non-study COVID-19 vaccinations. The period up to EUA vaccination, regardless of unblinding, is used to evaluate long term immunogenicity in AZD1222 participants. Participants who tested positive for SARS-CoV-2 nucleocapsid antibodies at any timepoint were excluded from exploratory *post-hoc* correlative analyses of antibody levels in serum and NELF (Figures 3 and S2). Immunogenicity substudy participants who did not receive dose 2 of the study intervention were excluded from timepoints following planned dose 2 administration date.

GMT and GMFR were calculated for AZD1222 and placebo groups and were summarized at each scheduled visit for anti-SARS-CoV-2 spike, receptor binding domain (RBD) and nucleocapsid antibodies by sample type (serum and NELF) and antibody isotype (i.e., IgG and IgA). Descriptive statistics for scheduled visits included GMTs, GMFRs, the number of participants, geometric mean, 95% confidence interval (CI), medians, minimum, and maximum. Similarly, GMTs were calculated for AZD1222 and placebo groups for participants with RT-PCR-confirmed SARS-CoV-2 infection ≥15 days following second dose primary series AZD1222 or placebo and were summarized at each illness visit for NELF anti-SARS-CoV-2 spike IgG and IgA antibodies. Descriptive statistics for illness visit analyses included GMTs, the number of participants, geometric mean, 95% CI, medians, minimum, and maximum. A GMT was calculated as the anti-logarithm of Σ(log base 2-transformed titer/n), i.e., as the anti-logarithm transformation of the mean of the log-transformed titer, where n is the number of participants with titer information. The 95% CI was calculated as the anti-logarithm transformation of the upper and lower limits for a two-sided CI for the mean of the log-transformed titers. A fold-rise was calculated as the ratio of the post-vaccination titer level to the pre-vaccination titer level. A GMFR was calculated as the anti-logarithm of Σ(log base 2-transformed (post-vaccination titer/pre-vaccination titer)/n). The 95% CI for a GMFR was calculated similarly to that for a GMT. The long-term nasal IgG immunogenicity data (Figure S1) were generated from a preliminary analysis for an early assessment of long-term immunogenicity up to day 360. These data were provided after the 6-month clinical database lock, and therefore have not undergone full reconciliation against the clinical database. NELF sample results received post-database lock are included for samples collected up to the 30 July 2021 data cut-off (Figure 4, Figure 5, Figure 6). Results received post-database lock have not been reconciled with the clinical database and therefore updates to these data may be applied in future analyses.

GMT and GMFR endpoints were analyzed using an analysis of variance (ANOVA) model that included the log base 2-transformed titer (GMT) or log base 2-transformed fold-rise (GMFR) as the dependent variable and study arm and age group as independent variables. On the log scale, the models were used to estimate a mean response for the AZD1222 and placebo groups and the difference (AZD1222 – placebo), with corresponding 95% confidence limits. These values were then back-transformed to give geometric means for the AZD1222 and placebo groups and a ratio of geometric means (AZD1222/placebo), with corresponding 95% confidence limits. A p-value, corresponding to a 2-sided test, was presented to compare AZD1222 against placebo. The p-value was nominal as exploratory endpoints were not controlled for multiplicity. This analysis was performed on participants who were seronegative at baseline (i.e., participants having a titer value < LLOQ at baseline) and was also performed separately by baseline serostatus.

Seroresponse is a binary outcome in which a success is defined as a fold-rise in titer compared to baseline of ≥4. Seroresponse rate was calculated for the AZD1222 and placebo groups and was summarized at each scheduled post-baseline visit for NELF anti-SARS-CoV-2 spike, RDB, and nucleocapsid antibodies by antibody isotype (IgG, IgA). The number and percentage of participants with post-vaccination seroresponse, and 95% CIs, were provided; the 95% CI of seroresponse rate was then calculated using the Clopper-Pearson exact method. These seroresponse summaries were also performed separately by baseline serostatus.

SAS 9.4 procedure SGPANEL was used to create the scatterplots for the correlative analyses (Figures 3, 5, 6, and S2). The REG statement generated the fitted regression line along with confidence limit intervals (CLI) and confidence limit for the mean (CLM) options to create the prediction limits and confidence limits respectively.

Statistical evidence between groups was determined by post-hoc two-tailed Mann-Whitney tests and categorized as: not significant, p > 0.05; ∗p ≤ 0.05; ∗∗p ≤ 0.01; ∗∗∗p ≤ 0.001.

The serum-to-NELF partition ratios, expressed as percentages, were computed as [(Nasal IgG MSD AU/mL x Dilution factor x MSD to AZ conversion factor x 100%)/serum IgG PPD AU/mL]. Partition ratios were determined for dilution factors based on the median, first, and third quartiles (Table S5).

### Additional resources

Further details on participating sites can be accessed via the study’s clinicaltrials.gov registry page: https://clinicaltrials.gov/study/NCT04516746.^23^

## Data Availability

• This paper does not report original code. • Any additional information required to reanalyze the data reported in this work paper is available from the lead contact upon request.

## Supplemental information

Document S1. Figures S1–S6 and Tables S1–S6

Table S3. Summary of GMTs, GMFRs, and seroresponse rates of anti-SARS-CoV-2 spike IgG and IgA as measured by MSD serology assay, related to Figures 1 and 2

Document S2. Article plus supplemental information
