# Supplementary material for: AZD1222-induced nasal antibody responses are shaped by prior SARS-CoV-2 infection and correlate with virologic outcomes in breakthrough infection
Source: Cell Rep Med. 2022 Dec 15;4(1):100882. doi: 10.1016/j.xcrm.2022.100882 (PMC9750884; doi:10.1016/j.xcrm.2022.100882)
Supplement: Document S1. Figures S1–S6 and Tables S1–S6 [file mmc1.pdf]

Cell Reports Medicine, Volume *4*

Supplemental information

AZD1222-induced nasal antibody responses are shaped by prior SARS-CoV-2 infection and correlate with virologic outcomes in breakthrough infection

Anastasia A. Aksyuk, Himanshu Bansal, Deidre Wilkins, Ann Marie Stanley, Stephanie Sproule, Jill Maaske, Satya Sanikommui, William R. Hartman, Magdalena E. Sobieszczyk, Ann R. Falsey, and Elizabeth J. Kelly

Supplemental information:

AZD1222-induced mucosal immune responses are shaped by prior SARS-CoV-2 infection and correlate with virologic outcomes in breakthrough infection

Anastasia A. Aksyuk, Himanshu Bansal, Deidre Wilkins, Ann Marie Stanley, Stephanie Sproule, Jill Maaske, Satya Sanikommui, William R. Hartman, Magdalena E. Sobieszczyk, Ann R. Falsey, Elizabeth J. Kelly

Supplemental Figures:

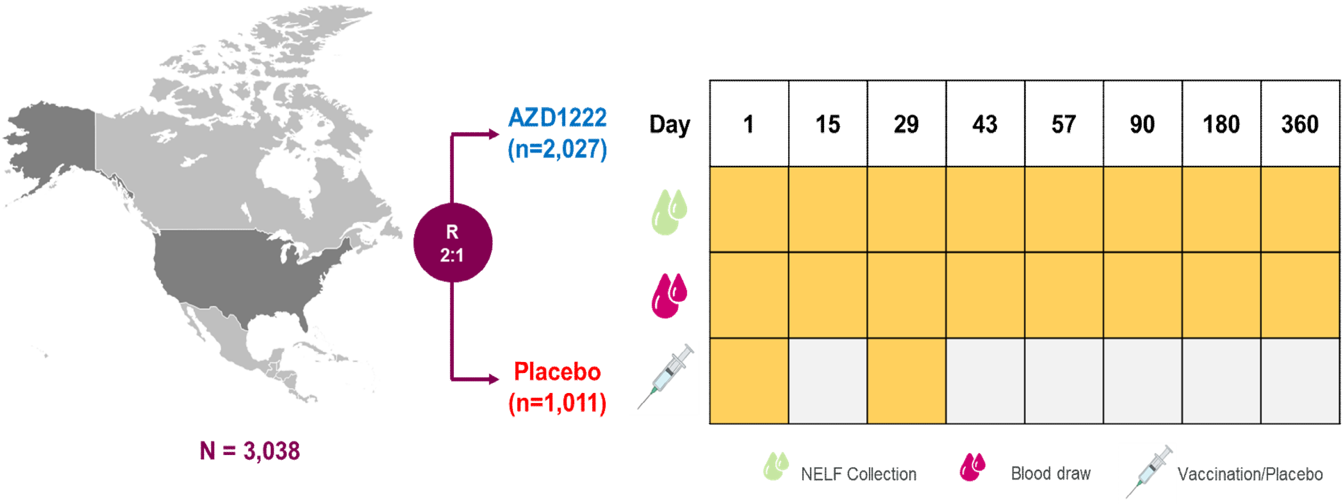

Supplemental Figure 1. Overview of the sample collection schedule for participants in the AZD1222 US/Chile/Peru phase 3 (NCT04516746) immunogenicity substudy. Related to Figures 1–3 and S2–4.

Schematic representation of sample collection schedule for NCT04516746 immunogenicity substudy participants. Yellow shading designates study days on which participant samples were obtained and AZD1222/placebo was administered. On days 1 and 29, NELF and serum samples were collected prior to the administration of AZD1222 or placebo. Immunogenicity data were windowed according to the timing of the first and second AZD1222/placebo doses to appropriately reflect the timepoint relative to the dosing days

NELF, nasal epithelium lining fluid.

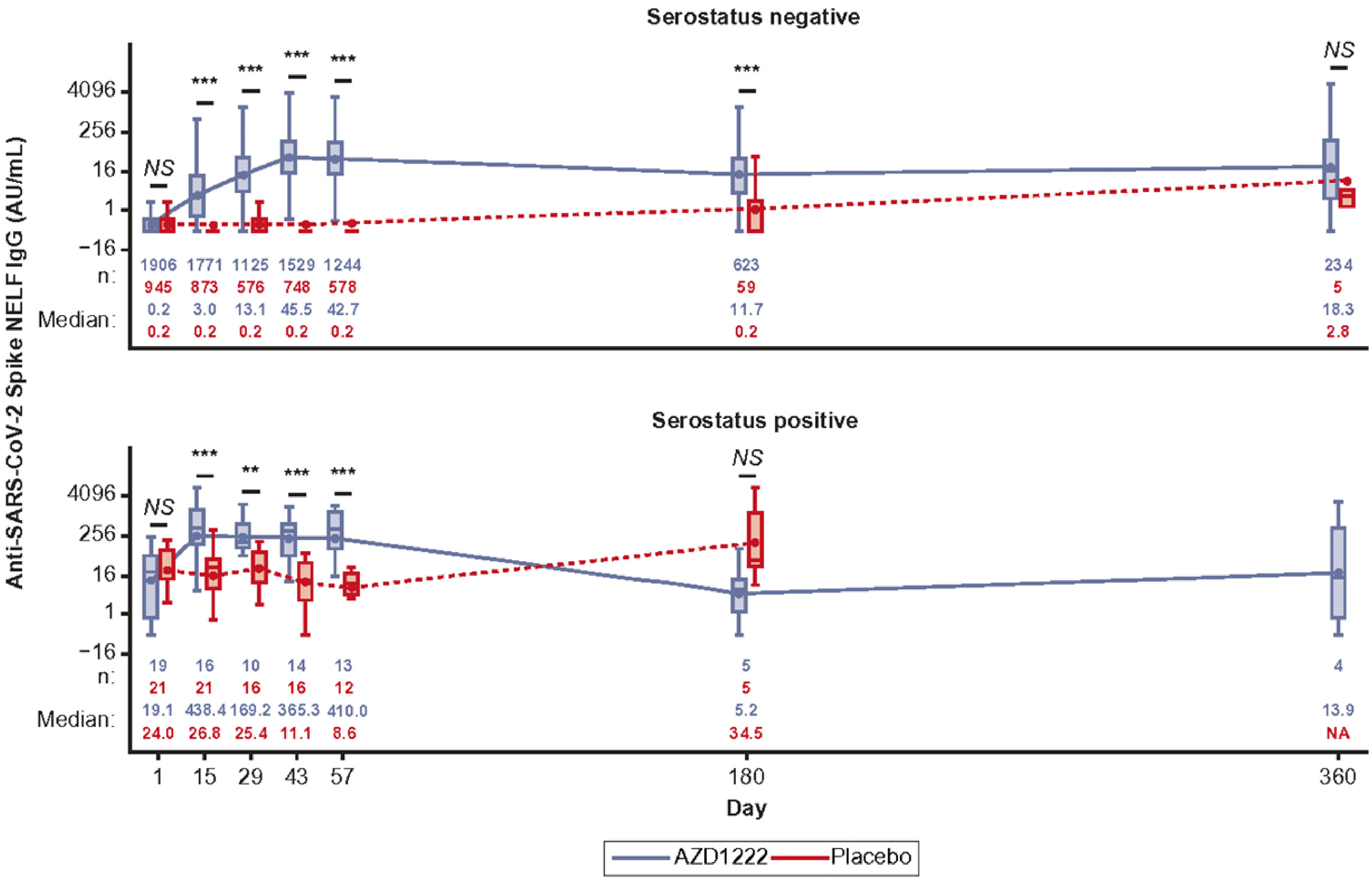

Serostatus negative
Anti-SARS-CoV-2 Spike NELF IgG (AU/mL)
4096
256
16
1
−16
NS
***
***
***
***
***
NS
n:
1906 1771 1125 1529 1244 623 234
945 873 576 748 578 59 5
Median:
0.2 3.0 13.1 45.5 42.7 11.7 18.3
0.2 0.2 0.2 0.2 0.2 0.2 2.8
Serostatus positive
NS
***
**
***
***
NS
n:
19 16 10 14 13 5 4
21 21 16 16 12 5
Median:
19.1 438.4 169.2 365.3 410.0 5.2 13.9
24.0 26.8 25.4 11.1 8.6 34.5 NA
1 15 29 43 57 180 360
Day
AZD1222
Placebo

Supplemental Figure 2. Long-term assessment of geometric mean titers of anti-SARS-CoV-2 spike IgG in nasal epithelial lining fluid from immunogenicity substudy participants following AZD1222 vaccination or placebo, by baseline serostatus. Related to Figure 1.

Boxplots illustrating IgG titers observed in serum following AZD1222 vaccination. X-axis denotes days since first AZD1222 dose. Day 1 and day 29 samples were obtained prior to the administration of AZD1222.

The box denotes IQR, the horizontal inside line the box denotes median, the marker inside the box is the geometric mean titer. Any points >1.5×IQR from the box were considered outliers and are not displayed. The whiskers that extend from the box indicate the minimum and maximum after removing the outliers. Boxplots are created using the log-normal distribution.

To provide comprehensive information on durability of immunogenicity post vaccination, data were censored in AZD1222 study participants at time of non-study COVID-19 vaccination, and for placebo participants at the earlier of the time of non-study COVID-19 vaccination or unblinding, whichever occurred first. Data from the placebo group showed enrichment of anti-spike antibodies from day 180, suggesting potential unreported non-study COVID-19 vaccinations, despite censoring for SARS-CoV-2 infection or known receipt of non-study COVID-19 vaccination.

In order to provide the most comprehensive data on durability of the nasal immune response following AZD1222 vaccination, data collected at the data cut-off (July 30, 2021) but that were not recorded in the database at the time of database lock date (September 2, 2021) were also included in this analysis. These additional data included some additional samples that were taken at earlier timepoints (e.g., at day 43) and at the day 180 and 360 timepoints. These data therefore have not undergone full reconciliation against the clinical database.

Statistical evidence between groups was determined by post-hoc two-tailed Mann-Whitney tests. NS, $p>0.05$; *$p\leq0.05$; **$p\leq0.01$; ***$p\leq0.001$.

AU/mL, arbitrary units per milliliter; IgG, immunoglobulin G; IQR, interquartile range; NELF, nasal epithelial lining fluid; NS, not significant.

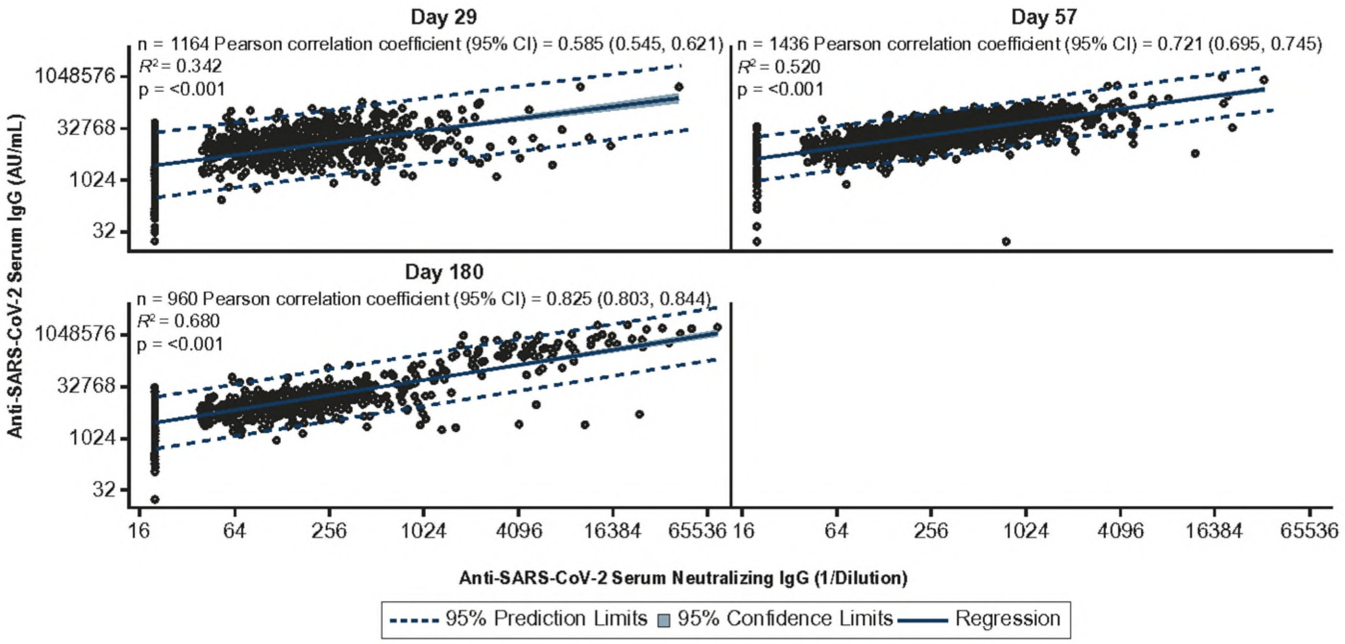

Supplemental Figure 3. Analysis of anti-SARS-CoV-2 spike and neutralizing IgG levels in serum from baseline-seronegative participants following AZD1222 vaccination. Related to Figure 1 and S1.

Post-hoc correlation analyses depicting the relationship between anti-spike and virus neutralizing IgG levels in serum obtained from baseline-seronegative participants in the immunogenicity substudy following AZD1222 vaccination or placebo. Clustering of participants along the y-axis occurs due to levels of serum anti-SARS-CoV-2 neutralizing IgG falling below the assay LLOQ. Lower limit of quantification (LLOQ) = 40 ID_{50}. 50% of LLOQ =20 ID_{50}.

AU/mL, arbitrary units per milliliter; CI, confidence interval; IgG, immunoglobulin G; LLOQ, lower limit of quantification.

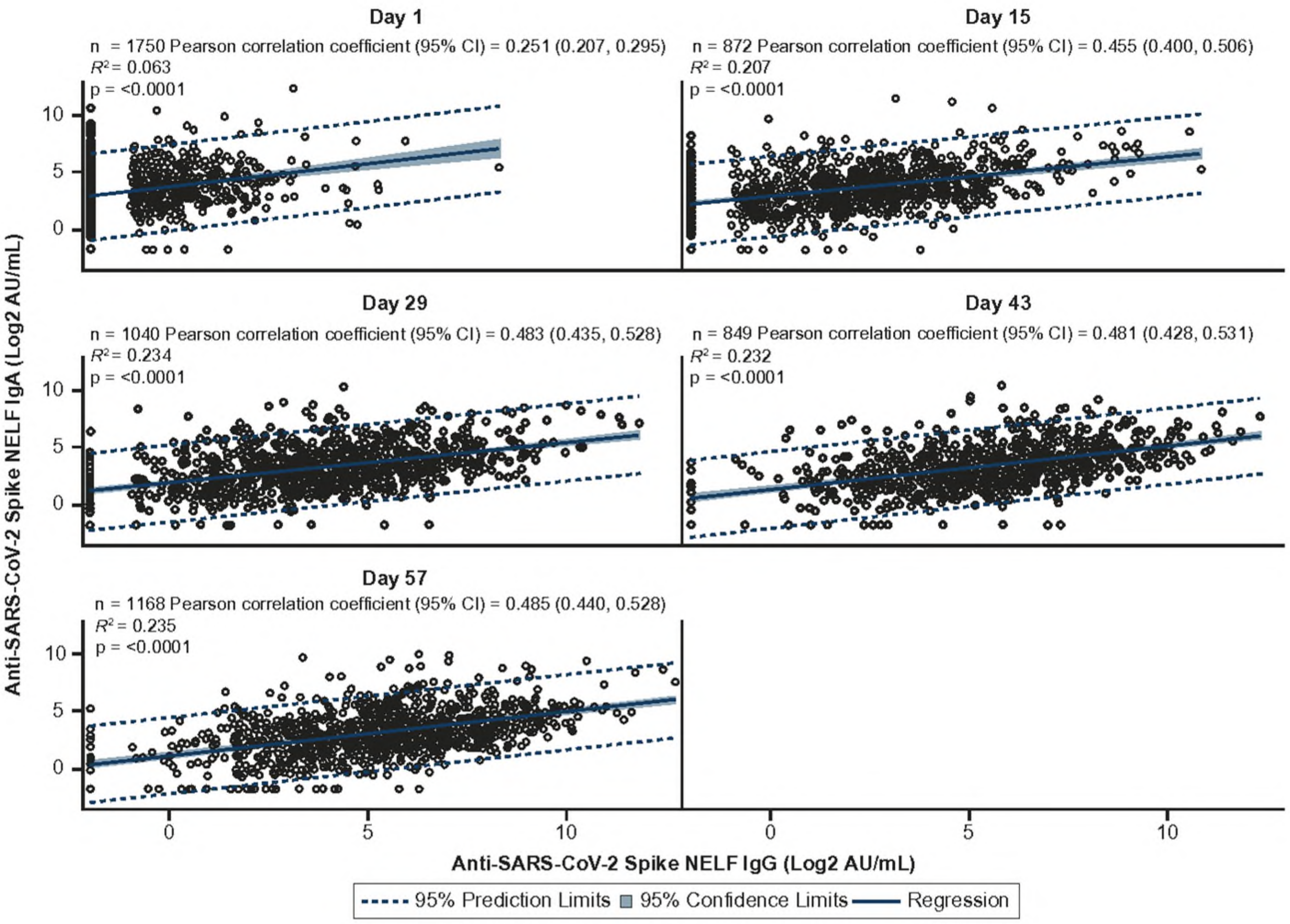

Supplemental Figure 4. Analysis of anti-SARS-CoV-2 spike IgA and IgG levels in baseline-seronegative immunogenicity substudy participant nasal epithelial lining fluid following AZD1222 vaccination. Related to Figure 3.

Scatterplot depicting the relationship between nasal anti-spike IgA levels (y-axis) and nasal anti-spike IgG levels (x-axis) following AZD1222 vaccination. Blue shading denotes 95% confidence limits. Dotted line denotes 95% prediction limits.

To provide comprehensive information on durability of immunogenicity post vaccination, data were censored in AZD1222 study participants at time of non-study COVID-19 vaccination, and for placebo participants at the earlier of the time of non-study COVID-19 vaccination or unblinding, whichever occurred first. Participants who tested positive for the presence of SARS-CoV-2 nucleocapsid antibodies at any time post-day 1 were excluded from this analysis.

AU/mL, arbitrary units per milliliter; CI, confidence interval; IgA/G, immunoglobulin A/G; NELF, nasal epithelial lining fluid.

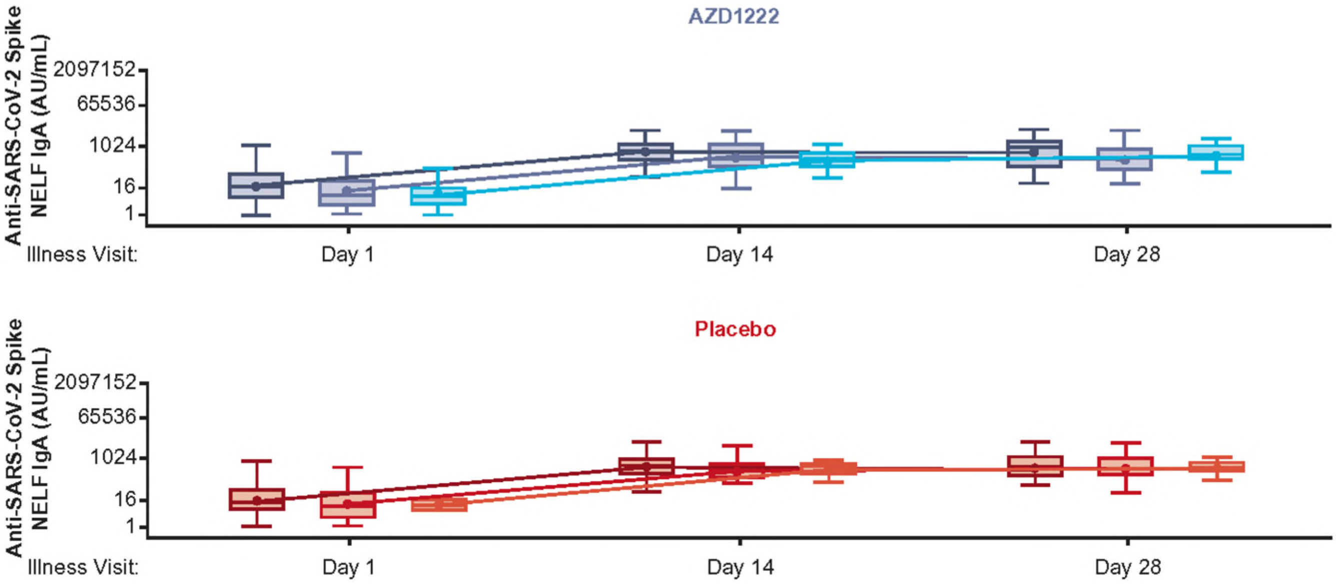

Illness Visit		Day 1		Day 14		Day 28	
		n	Median	n	Median	n	Median
AZD1222	<60 Days	67	15.4	37	638.0	20	765.4
	60–120 Days	44	7.5	45	283.0	40	264.1
	>120 Days	15	7.0	15	259.0	13	390.0
Placebo	<60 Days	96	12.0	61	455.5	34	461.0
	60–120 Days	45	8.3	36	314.7	38	384.8
	>120 Days	2	9.2	5	415.0	6	376.4

< 60 Days 60–120 Days > 120 Days

Supplemental Figure 5. Quantification of anti-SARS-CoV-2 spike IgA levels in nasal epithelial lining fluid from study participants with symptomatic breakthrough SARS-CoV-2 infection by time since second dose primary series vaccination or placebo. Related to Figure 4.

Boxplots illustrating anti-SARS-CoV-2 spike IgA titers observed in NELF obtained from baseline-seronegative study participants following RT-PCR-positive symptomatic breakthrough SARS-CoV-2 infection ≥15 days post-second AZD1222 vaccination or placebo. Results are presented by time since second dose primary series AZD1222 or placebo (i.e., <60 days, 60–120 days and >120 days). X-axis denotes days since the first illness visit for a period of 28 days.

The box denotes IQR, the horizontal line inside the box denotes median, the marker inside the box is the geometric mean titer. Any points >1.5×IQR from the box were considered outliers and are not displayed. The whiskers that extend from the box indicate the minimum and maximum after removing the outliers. Boxplots are created using the log-normal distribution. IgA values between 0 and 1 are imputed as 1 to avoid negative log values.

Participants who have been unblinded or received non-study COVID-19 vaccination or exclusionary medication have been excluded from this analysis. NELF sample results received post-database lock are included for samples collected up to the 30 July 2021 data cut-off. Results received post-database lock have not been reconciled with the clinical database and therefore updates to these data may be applied.

AU/mL, arbitrary units per milliliter; IgA, immunoglobulin A; IQR, interquartile range; NELF, nasal epithelial lining fluid; RT-PCR, reverse transcription polymerase chain reaction.

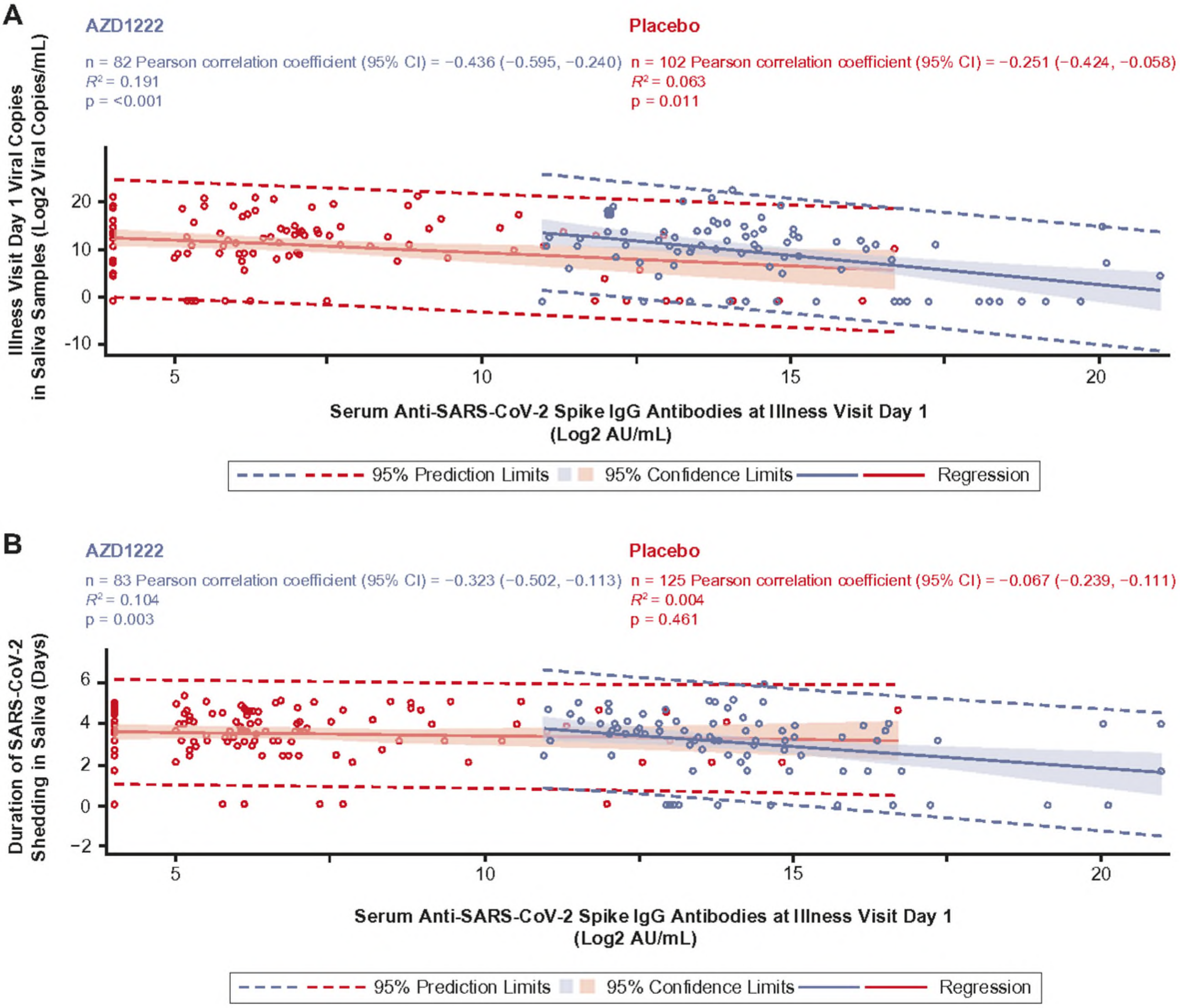

Supplemental Figure 6. Analysis of Illness Visit Day 1 anti-SARS-CoV-2 spike IgG levels in serum versus Illness Visit Day 1 viral load (A) and duration of viral shedding (B) in saliva samples from study participants with symptomatic breakthrough SARS-CoV-2 infection ≥15 days post-AZD1222 primary series dose 2 or placebo. Related to Figure 5.

(A–B) Post-hoc correlation analyses depicting the relationship between Illness Visit Day 1 anti-spike IgG levels in serum (x-axes) versus Illness Visit Day 1 viral load in saliva samples (A) and duration of viral shedding in saliva samples (B) obtained from baseline-seronegative study participants with RT-PCR-positive symptomatic breakthrough SARS-CoV-2 infection ≥15 days post-second AZD1222 vaccination or placebo. Shading denotes 95% confidence limits. Dotted line denotes 95% prediction limits.

Participants who had been unblinded or received non-study COVID-19 vaccination or exclusionary medication are excluded from this analysis.

AU/mL, arbitrary units per milliliter; CI, confidence interval; IgG, immunoglobulin G; RT-PCR, reverse transcription polymerase chain reaction.

Supplemental Tables:

Supplemental Table 1. Baseline characteristics of participants in the immunogenicity substudy cohort. Related to STAR methods.

	**AZD1222 (n = 2,027)**	**Placebo (n = 1,011)**	**Total (N = 3,038)**
**Sex and age**			
Female, n (%)	814 (40.2)	430 (42.5)	1,244 (40.9)
Male, n (%)	1,213 (59.8)	581 (57.5)	1,794 (59.1)
Mean age (SD)	55.0 (15.82)	54.4 (16.40)	54.8 (16.01)
Median age (min–max)	55.0 (18–100)	55.0 (18–90)	55.0 (18–100)
**Age, n (%)**			
≥18 to <56 years	1,014 (50.0)	513 (50.7)	1,527 (50.3)
≥56 to <70 years	523 (25.8)	246 (24.3)	769 (25.3)
≥70 years	490 (24.2)	252 (24.9)	742 (24.4)
**Ethnicity, n (%)**			
Hispanic or Latinx	170 (8.4)	87 (8.6)	257 (8.5)
Not Hispanic or Latinx	1,809 (89.2)	911 (90.1)	2,720 (89.5)
Not reported	43 (2.1)	13 (1.3)	56 (1.8)
Unknown	5 (0.2)	0	5 (0.2)
**Race, n (%)**			
Multiple[a]	26 (1.3)	11 (1.1)	37 (1.2)
Asian	53 (2.6)	19 (1.9)	72 (2.4)
Black or African American	105 (5.2)	59 (5.8)	164 (5.4)
American Indian or Alaska Native	13 (0.6)	10 (1.0)	23 (0.8)
Native Hawaiian or Pacific Islander	3 (0.1)	1 (0.1)	4 (0.1)
White	1,804 (89.0)	902 (89.2)	2,706 (89.1)

Not reported	11 (0.5)	6 (0.6)	17 (0.6)
Unknown	12 (0.6)	3 (0.3)	15 (0.5)
**Infection status at baseline,[b] n (%)**			
Naïve	1,984 (97.9)	975 (96.4)	2,959 (97.4)
Prior SARS-CoV-2	20 (1.0)	22 (2.2)	42 (1.4)
Missing	8 (0.4)	3 (0.3)	11 (0.4)
Not done	15 (0.7)	11 (1.1)	26 (0.9)
**COVID-19 comorbidities[c] n (%)**			
Yes	1,279 (63.1)	655 (64.8)	1,934 (63.7)
No	748 (36.9)	356 (35.2)	1,104 (36.3)

[a]Participants who reported more than one race are reported under 'Multiple'. [b]Serostatus at baseline is defined by the nucleocapsid antibody level as measured by Roche Elecsys Anti-SARS-CoV-2 serology test. [c]Conditions which place subject at high risk for acquisition or more severe COVID-19 disease defined as per (Falsey et al., 2021).

SD, standard deviation.

Supplemental Table 2. Dosing interval per time of enrolment in relation to clinical hold. Related to STAR methods.

**Dosing interval, days**	**AZD1222**	**Placebo**
**Overall, n**	1,952	968
Mean (SD)	37.5 (14.34)	37.7 (14.89)
Median (min–max)	29.0 (21–148)	29.0 (23–154)
**Participants enrolled prior to clinical hold, n**	516	259
Mean (SD)	60.6 (5.72)	60.7 (7.51)
Median (min–max)	60.0 (53–148)	59.0 (54–154)
**Participants enrolled post clinical hold, n**	1,436	709
Mean (SD)	29.2 (2.51)	29.2 (4.07)
Median (min–max)	29.0 (21–63)	29.0 (23–126)

The participants randomized prior to the clinical hold are those who received their first dose of study intervention between 28 August 2020 and 06 September 2020. In order to be included in this summary of dosing intervals, substudy participants must have received both doses of AZD1222 or placebo. The dosing interval (in days) is calculated as the date of dose 2 – date of dose 1 +1.

SD, standard deviation.

Supplemental Table 4. Demographics of participants with breakthrough SARS-CoV-2 infection ≥15 days post-AZD1222 primary series dose 2 or placebo. Related to Figure 4.

	**AZD1222 (n = 177)[a]**		**Placebo (n = 203)[a]**		**Total (N = 380)[a]**	
	**≥18–<65 years (n = 167)**	**≥65 years (n = 10)**	**≥18–<65 years (n = 181)**	**≥65 years (n = 22)**	**≥18–<65 years (n = 348)**	**≥65 years (n = 32)**
**Sex and age**						
Female, n (%)	65 (38.9)	5 (50.0)	69 (38.1)	6 (27.3)	134 (38.5)	11 (34.4)
Male, n (%)	102 (61.1)	5 (50.0)	112 (61.9)	16 (72.7)	214 (61.5)	21 (65.6)
Mean age (SD)	39.82 (11.860)	71.50 (6.329)	43.67 (12.948)	69.50 (4.657)	41.82 (12.569)	70.13 (5.216)
Median age (min–max)	41.0 (18, 64)	69.0 (66, 85)	44.0 (18, 64)	68.5 (65, 83)	42.5 (18, 64)	69.0 (65, 85)
**Age, n (%)**						
≥18 to <65 years	167 (100)	-	181 (100)	-	348 (100)	-
≥65 to <75 years	-	7 (70.0)	-	20 (90.9)	-	27 (84.4)
≥75 years	-	3 (30.0)	-	2 (9.1)	-	5 (15.6)
**Ethnicity, n (%)**						
Hispanic or Latinx	70 (41.9)	1 (10.0)	59 (32.6)	6 (27.3)	129 (37.1)	7 (21.9)
Not Hispanic or Latinx	95 (56.9)	9 (90.0)	120 (66.3)	16 (72.7)	215 (61.8)	25 (78.1)
Not reported	2 (1.2)	-	2 (1.1)	-	4 (1.1)	-
**Race, n (%)**						
Multiple[b]	11 (6.6)	-	13 (7.2)	1 (4.5)	24 (6.9)	1 (3.1)
Asian	6 (3.6)	-	4 (2.2)	-	10 (2.9)	-
Black or African American	7 (4.2)	-	15 (8.3)	1 (4.5)	22 (6.3)	1 (3.1)
American Indian or Alaska Native	29 (17.4)	1 (10.0)	18 (9.9)	3 (13.6)	47 (13.5)	4 (12.5)
Native Hawaiian or Pacific Islander	2 (1.2)	-	-	-	2 (0.6)	-
White	112 (67.1)	9 (90.0)	127 (70.2)	17 (77.3)	239 (68.7)	26 (81.3)

Not reported	-	-	4 (2.2)	-	4 (1.1)	-
**Country, n (%)**						
USA	114 (68.3)	9 (90.0)	147 (81.2)	18 (81.8)	261 (75.0)	27 (84.4)
Chile	11 (6.6)	-	10 (5.5)	-	21 (6.0)	-
Peru	42 (25.1)	1 (10.0)	24 (13.3)	4 (18.2)	66 (19.0)	5 (15.6)
**COVID-19 serostatus at baseline**						
Negative	167 (100)	10 (100)	181 (100)	22 (100)	348 (100)	32 (100)
**COVID-19 comorbidities[c] n (%)**						
Yes	85 (50.9)	8 (80.0)	109 (60.2)	17 (77.3)	194 (55.7)	25 (78.1)
No	82 (49.1)	2 (20.0)	72 (39.8)	5 (22.7)	154 (44.3)	7 (21.9)

[a]Number of participants who initiated illness visits. However, not all ill participants contributed data to every illness visit. [b]Participants who reported more than one race are reported under 'Multiple'. [c]Conditions which place subject at high risk for acquisition or more severe COVID19 disease defined as per [1]. SD, standard deviation.

Supplemental Table 5. Summary of serum: NELF IgG partition ratio (%) using first quartile (3.38) and third quartile (8.19) dilution factors in baseline-seronegative substudy participants following AZD1222 vaccination. Related to Table 1.

	First quartile (3.38) dilution factor					Third quartile (8.19) dilution factor				
**Summary statistics**	**Day 1**	**Day 15**	**Day 29**	**Day 43**	**Day 57**	**Day 1**	**Day 15**	**Day 29**	**Day 43**	**Day 57**
n	1,875	903	1,101	899	1,212	1,875	903	1,101	899	1,212
Partition ratio geometric mean	3.05	0.72	0.84	0.74	0.81	7.41	1.74	2.03	1.80	1.96
95% CI for geometric mean	(2.92, 3.20)	(0.66, 0.78)	(0.77, 0.91)	(0.68, 0.81)	(0.75, 0.88)	(7.07, 7.77)	(1.59, 1.90)	(1.87, 2.20)	(1.64, 1.97)	(1.81, 2.13)
Geometric %CV	1.38	2.29	2.35	2.48	2.62	1.38	2.29	2.35	2.48	2.62
Min	0.03	0.00	0.01	0.00	0.00	0.08	0.01	0.01	0.00	0.01
Max	176.79	33.48	178.85	34.91	9,528.80	428.91	81.21	433.89	84.69	23,117.17

CI, confidence interval; CV, coefficient of variation; IgG, immunoglobulin G; NELF, nasal epithelial lining fluid.

In order to provide comprehensive information on durability of immunogenicity post vaccination, data was censored in study participants at time of receipt of non-study COVID-19 vaccine, if applicable, but not at time of unblinding.

Supplemental Table 6: Evidence for potential unreported non-study COVID-19 vaccinations in baseline-seronegative and baseline-seropositive participants in the placebo group with SARS-CoV-2 spike antibody titers (IgG) in nasal secretions.[a,b] Related to STAR Methods.

Post-baseline visit	Participants with valid baseline and post-baseline visit result, n	Participants with ≥4-fold increase from baseline in spike IgG but not nucleocapsid IgG titer, n (%)
**Baseline-seronegative participants**		
Any	910	23 (2.5)
Day 15	827	5 (0.6)
Day 29	546	4 (0.7)
Day 43	712	7 (1.0)
Day 57	539	5 (0.9)
Day 180	50	6 (12.0)
**Baseline-seropositive participants**		
**Post-baseline visit**	**Participants with valid baseline and post-baseline visit result, n**	**Participants with increase from baseline in spike IgG but not nucleocapsid IgG titer, n (%)**
Any	20	6 (30.0)
Day 15	20	4 (20.0)
Day 29	15	0
Day 43	14	0
Day 57	11	1 (9.1)
Day 180	20	6 (30.0)

[a]Data were censored for placebo recipients at the time of non-study COVID-19 vaccination or unblinding or symptomatic illness, whichever occurred earlier. An additional censoring criterion of a positive nucleocapsid test was used for baseline-seronegative participants only. [b]Participants without a baseline result are excluded from these tables but are included in Figures 1, 2 and S1 if they had a valid post-baseline result.

References:

1. Falsey, A.R., Sobieszczyk, M.E., Hirsch, I., Sproule, S., Robb, M.L., Corey, L., Neuzil, K.M., Hahn, W., Hunt, J., Mulligan, M.J., et al. (2021). Phase 3 safety and efficacy of AZD1222 (ChAdOx1 nCoV-19) Covid-19 vaccine. N Engl J Med *385*, 2348-2360. 10.1056/NEJMoa2105290.
